# The Genetic and Biochemical Basis of FANCD2 Monoubiquitination

**DOI:** 10.1016/j.molcel.2014.05.001

**Published:** 2014-06-05

**Authors:** Eeson Rajendra, Vibe H. Oestergaard, Frédéric Langevin, Meng Wang, Gillian L. Dornan, Ketan J. Patel, Lori A. Passmore

**Affiliations:** 1 MRC Laboratory of Molecular Biology, Francis Crick Avenue, Cambridge CB2 0QH, UK; 2 Department of Medicine, Level 5, Addenbrooke’s Hospital, University of Cambridge, Cambridge CB2 0QQ, UK

## Abstract

Fanconi anaemia (FA) is a cancer predisposition syndrome characterized by cellular sensitivity to DNA interstrand crosslinkers. The molecular defect in FA is an impaired DNA repair pathway. The critical event in activating this pathway is monoubiquitination of FANCD2. In vivo, a multisubunit FA core complex catalyzes this step, but its mechanism is unclear. Here, we report purification of a native avian FA core complex and biochemical reconstitution of FANCD2 monoubiquitination. This demonstrates that the catalytic FANCL E3 ligase subunit must be embedded within the complex for maximal activity and site specificity. We genetically and biochemically define a minimal subcomplex comprising just three proteins (FANCB, FANCL, and FAAP100) that functions as the monoubiquitination module. Residual FANCD2 monoubiquitination activity is retained in cells defective for other FA core complex subunits. This work describes the in vitro reconstitution and characterization of this multisubunit monoubiquitin E3 ligase, providing key insight into the conserved FA DNA repair pathway.

## Highlights

• A native Fanconi anaemia core complex is purified from vertebrate cells
• Purified FA core complex monoubiquitinates FANCD2 in vitro
• FANCB, FANCL, and FAAP100 constitute the minimal monoubiquitination module
• Other subunits contribute to maximal activity and site specificity of FANCL

## Graphical Abstract

**Figure:**
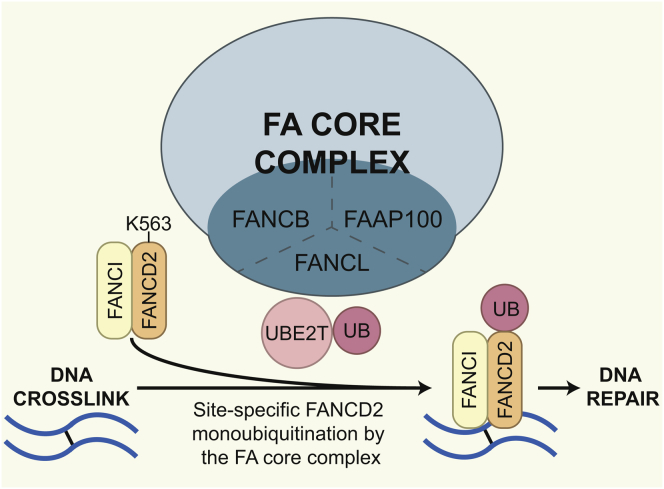

## Teaser

FANCD2 monoubiquitination by the multisubunit Fanconi anaemia (FA) core complex is the key activating event in a conserved DNA repair pathway. Rajendra et al. now purify the core complex and reconstitute the monoubiquitination event in vitro. Genetic and biochemical dissection define a three-component subcomplex that functions as the catalytic monoubiquitination module.

## Introduction

Fanconi anaemia (FA) is a rare, recessive disorder with a striking phenotype consisting of developmental defects, bone marrow failure, and cancer predisposition (Crossan and Patel, 2012). At the heart of FA are deficiencies in a network of proteins that constitute a conserved eukaryotic DNA repair pathway that targets DNA interstrand crosslinks and counteracts aldehyde genotoxicity (Kim and D’Andrea, 2012; Langevin et al., 2011). FA patients harbor inactivating mutations in any one of 15 *FANC* genes (Kottemann and Smogorzewska, 2013). In addition, five FA-associated proteins (FAAPs) play roles in the FA DNA repair pathway.

A number of FANC and FAAP proteins (FANCA, FANCB, FANCC, FANCE, FANCF, FANCG, FANCL, and FANCM and FAAP20, FAAP24, and FAAP100) associate to form the FA core complex (Hodson and Walden, 2012; Meetei et al., 2003b). After DNA damage, this multisubunit E3 ligase carries out the specific monoubiquitination of FANCI and FANCD2, a critical event that initiates DNA repair (Garcia-Higuera et al., 2001; Knipscheer et al., 2009; Smogorzewska et al., 2007). The sites of monoubiquitination have been mapped to the conserved lysine 561 in human FANCD2 (K563 in chicken) and lysine 523 in human FANCI (K525 in chicken) (Garcia-Higuera et al., 2001; Ishiai et al., 2008; Matsushita et al., 2005; Sims et al., 2007; Smogorzewska et al., 2007). Ubiquitinated FANCD2 is a platform for the recruitment of additional proteins that coordinate DNA repair. These include the FAN1 nuclease and the scaffold protein FANCP (SLX4), which recruits the XPF-ERCC1, SLX1, and MUS81-EME1 nucleases (Cybulski and Howlett, 2011; Huang and D’Andrea, 2010).

Mechanistic insight into the FA core complex is lacking because it has yet to be purified in a form suitable for in vitro biochemical characterization. Challenges stem from the large number of subunits, their low abundance, and the absence of orthologs in lower eukaryotic model organisms such as yeast (Zhang et al., 2009). Other multisubunit E3 ligases, such as the anaphase-promoting complex/cyclosome (APC/C) and Skp-Cullin-F box (SCF) complex, function by precisely positioning substrate proteins for ubiquitination by their really interesting gene (RING) finger subunits (Zimmerman et al., 2010). Their complex architectures allow extensive regulation. The mechanism of the FA core complex is of particular interest because of its monoubiquitin ligase function (contrasting with either APC/C or SCF, which form polyubiquitin chains), its central position in the FA DNA repair pathway and its fundamental role in human health.

In vitro assays have been reconstituted with the FANCL RING finger subunit in the absence of other FA core complex subunits (Alpi et al., 2008; Longerich et al., 2009; Sato et al., 2012). RING finger E3 ligases are known to specifically recruit E2 ubiquitin-conjugating enzymes in order to allow ubiquitin transfer from E2 onto a substrate. Ubiquitin conjugating enzyme 2T (UBE2T) functions as an E2 enzyme for FANCL both in vitro and in vivo (Alpi et al., 2008, 2007; Hodson et al., 2014; Machida et al., 2006). A second E2 (UBE2W) binds FANCL and monoubiquitinates FANCD2 in vitro (Alpi et al., 2008; Zhang et al., 2011), but its role in the FA pathway has not been rigorously explored. Recent experiments have also revealed that efficient recognition of FANCD2 by FANCL in vitro depends on the presence of its binding partner FANCI and DNA (Sato et al., 2012). The roles of other FA subunits are poorly defined, and they largely lack any identifiable domains that could illuminate their function.

Here, we develop a purification strategy for isolating a native FA core complex. The recovered complex is efficient at FANCD2 monoubiquitination in a cell-free system, and our biochemical analysis points to a core subcomplex as being fundamental to this reaction. Finally, genetic ablation studies confirm an essential role for components of this minimal subcomplex in vivo.

## Results

### Purification of a Native FA Core Complex

**Figure 1:**
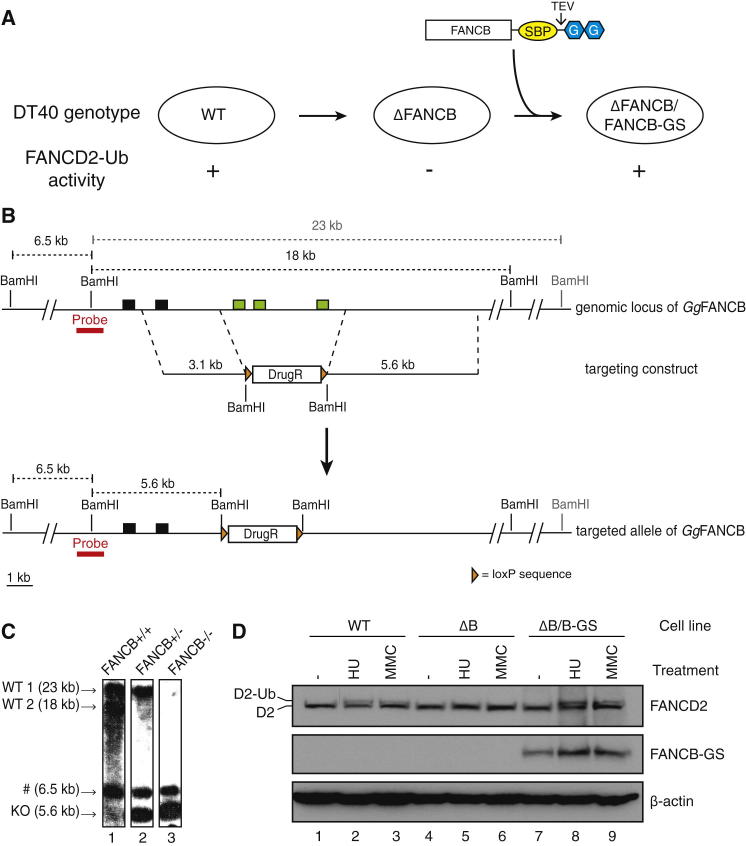
Generation and Validation of the Δ*FANCB*/*FANCB-GS* DT40 Cell Line (A) Schematic representation of generation of the Δ*FANCB*/*FANCB-GS* cell line and the expected effect on FANCD2 monoubiquitination in vivo. G, the IgG binding domain of protein G; SBP, streptavidin binding peptide. (B) Targeting strategy for the generation of the Δ*FANCB* cell line. Exons are shown in black (exons 5 and 6) or green (exons 7, 8, and 9) boxes. The location of the Southern blot probe is in red. DrugR, drug resistance cassette. (C) Southern blot showing gene targeting of *FANCB*. After *BamH*I digest, wild-type (WT) alleles are 23 or 18 kb, whereas the knockout allele (KO) is 5.6 kb. A 6.5 kb fragment (#) is present in all cell lines. Lane 1 shows a *FANCB*+/+ cell line, lane 2 shows *FANCB*+/−, and lane 3 shows *FANCB*–/–. (D) Δ*FANCB*/*FANCB-GS* cells regained the ability to monoubiquitinate FANCD2 upon treatment with 1 mM hydroxyurea (HU) or 50 ng/ml mitomycin C (MMC). Western blotting of FANCD2 monoubiquitination in whole-cell extracts is shown for WT DT40 cells, cells with a deletion of the genomic *FANCB* gene (ΔB), and ΔB cells stably expressing chicken *FANCB* with a C-terminal protein G and streptavidin binding peptide tag (ΔB/B-GS).

We developed a purification strategy to isolate the FA core complex from chicken DT40 cells, a vertebrate model used extensively in the study of the FA pathway (Niedzwiedz et al., 2004; Yamamoto et al., 2003). Our aim was to generate a cell line where the only copy of the FANCB subunit included an affinity purification tag (Figure 1A). First, we deleted the genomic copies of *FANCB* (Figures 1B and 1C). In wild-type cells, FANCD2 is monoubiquitinated after exposure to DNA damage or replication stress, for example, by the DNA crosslinking agent mitomycin C (MMC) or the DNA synthesis inhibitor hydroxyurea (HU). This can be monitored on western blots by the appearance of a slower migrating monoubiquitinated form of FANCD2 (Figure 1D, lanes 2 and 3). Δ*FANCB* DT40 cells have a defective FA pathway because they do not exhibit FANCD2 monoubiquitination after MMC or HU treatment (Figure 1D, lanes 5 and 6), which is in agreement with previous work in FA patient cells that lack functional *FANCB* (Meetei et al., 2004).

Next, *FANCB* with a C-terminal protein G-streptavidin binding peptide (GS) tag (Bürckstümmer et al., 2006) was used to complement the Δ*FANCB* cells (Figure 1A). Δ*FANCB/FANCB-GS* cells regained the ability to monoubiquitinate FANCD2 (Figure 1D, lanes 8 and 9). Thus, the tagged FANCB protein is functional and restores the activity of the FA core complex in vivo.

**Figure 2:**
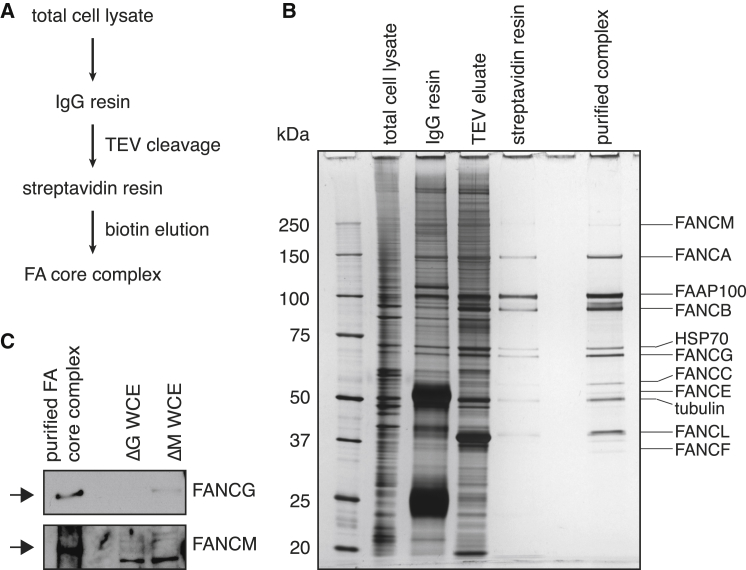
Purification of the Fanconi Anaemia Core Complex (A) FA core complex purification strategy. (B) SDS-PAGE and silver stain analysis of the purification of FA core complex from Δ*FANCB/FANCB-GS* cells. Major species identified by mass spectrometry (data not shown) are indicated. (C) The presence of FANCG and FANCM in purified FA core complex is confirmed by western blotting with antibodies against the endogenous proteins. The purified core complex was run on SDS-PAGE alongside whole-cell extracts (WCE) from Δ*FANCG* and Δ*FANCM* DT40 cells. See also Table S1.

Using the Δ*FANCB/FANCB-GS* DT40 cell line, we were able to purify the FA core complex with IgG agarose using elution by tobacco etch virus (TEV) cleavage and subsequent purification on streptavidin resin with biotin elution, as shown in Figure 2A. This yielded highly pure native FA core complex under physiological conditions. The purified complex contains nine known subunits whose identities were confirmed by western blotting and mass spectrometry: FANCA, FANCB, FANCC, FANCE, FANCF, FANCG, FANCL, FANCM, and FAAP100 (Figures 2B and 2C and data not shown). Quantitative SDS-PAGE analysis indicated that the subunits can be classified into three groups on the basis of their relative abundances: FANCB-FANCL-FAAP100 (B-L-100), FANCC-FANCE-FANCF (C-E-F), and FANCA-FANCG (A-G; Table S1 available online). The FANCM subunit is present in very low abundance, whereas its associated proteins (FAAP10, FAAP16, and FAAP24) and the FANCA-associated protein FAAP20 are not detected in our purification. These subunits are not essential for FANCD2 monoubiquitination or the structural integrity of the FA core complex in vivo but most likely facilitate chromatin localization (Kim et al., 2008; Mosedale et al., 2005; Singh et al., 2009, 2010; Yan et al., 2010). Thus, our purified FA core complex contains all subunits currently known to be required for FANCD2 monoubiquitination, demonstrating that a constitutively assembled core complex exists in asynchronous dividing cells independent of DNA damage (Alpi et al., 2007).

### In Vitro FANCD2 Monoubiquitination by Purified FA Core Complex

**Figure 3:**
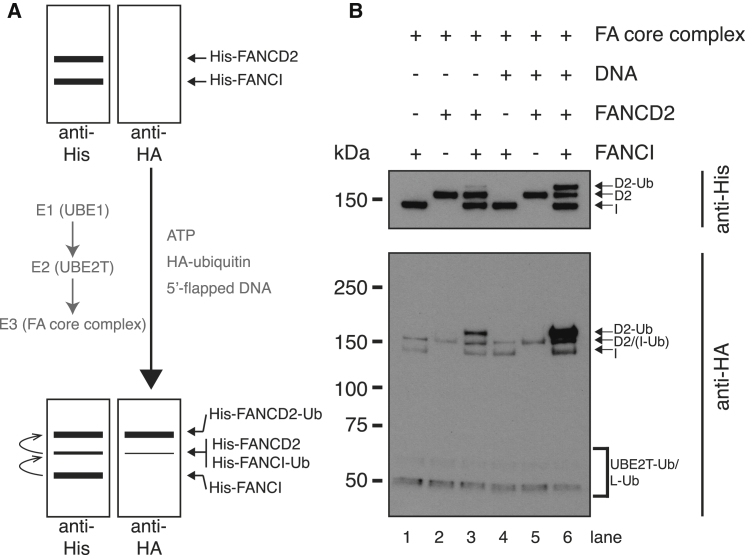
Purified FA Core Complex Monoubiquitinates FANCD2 in the Context of FANCI and DNA (A) Schematic of the in vitro ubiquitination assay. Assays were monitored by western blotting with anti-His to detect His-tagged FANCI and FANCD2 or anti-HA to detect HA-tagged ubiquitin. (B) Monoubiquitination of FANCD2 and the FANCI-FANCD2 complex in the absence or presence of 5′-flapped DNA after 90 min at 30°C. FANCI and FANCD2 show mild cross-reactivity with anti-HA. Autoubiquitination of FANCL and UBE2T are labeled. FA core complex concentration is ∼200 nM. See Figure S1D for analysis after an additional 12 hr at room temperature. See also Figure S1.

The E3 ligase in the FA core complex is the RING-finger-containing FANCL subunit (Alpi et al., 2008; Cole et al., 2010; Meetei et al., 2003a). The roles of other subunits are unclear. In vivo mutation of any one of the core complex components is thought to abolish FANCD2 monoubiquitination and lead to defective DNA repair and disease (Garcia-Higuera et al., 2001). To investigate the biochemical activity of the FA core complex, we reconstituted its monoubiquitination activity in vitro. We incubated purified FA core complex with recombinant E1 ubiquitin-activating enzyme, UBE2T, HA-tagged ubiquitin, and the His-tagged substrates FANCD2 and FANCI (Figures 3A and S1A–S1C). To eliminate species incompatibilities, we used the chicken homologs of UBE2T, FANCD2, and FANCI. We monitored monoubiquitination of His-FANCD2 and His-FANCI by western blotting.

In this assay, our purified FA core complex catalyzes the transfer of ubiquitin onto FANCD2, as shown by the appearance of a discrete slower migrating band above FANCD2 on both anti-His and anti-HA western blots in Figures 3B and S1D (lanes 3 and 6). The reaction goes to near completion at late time points, where unmodified FANCD2 is barely visible (Figure S1D, lane 6). Thus, our purified complex is active.

FANCD2 forms a complex in vivo with FANCI, and both are monoubiquitinated and recruited to sites of DNA damage in chromatin (Smogorzewska et al., 2007; Matsushita et al., 2005; Montes de Oca et al., 2005). We found that the in vitro activity of purified FA core complex is dependent on the presence of FANCI and is greatly stimulated by 5′-flapped DNA (Figure 3B), reflecting the in vivo context of its activity.

### The Intact FA Core Complex Is More Active and More Specific than Isolated FANCL

As mentioned above, all previous in vitro studies of FANCD2 monoubiquitination had been performed with isolated FANCL (Alpi et al., 2008; Sato et al., 2012). We sought to compare this with the activity of our purified complex (Figures S1E and S1F).

**Figure 4:**
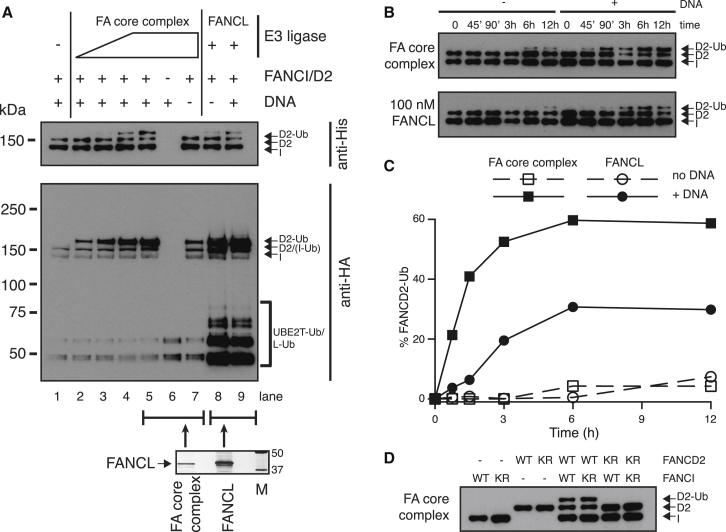
The FA Core Complex Has Higher Activity and Specificity than Isolated FANCL (A) Titration of the FA core complex in a 2-fold dilution series (lanes 2–5) is compared to isolated FANCL (lanes 8 and 9) in the monoubiquitination assay after 90 min at 30°C monitored by western blotting. See Figure S2A for analysis after an additional 12 hr at room temperature. The bottom panel is a silver-stained gel showing the relative amounts of isolated FANCL (1 μM) used for the reactions in lanes 8 and 9 and FANCL in the FA core complex (∼200 nM) at the highest concentration in lanes 5–7. The full gel is shown in Figure S2B. (B) Time course of FANCD2 monoubiquitination by the FA core complex (top) and a molar equivalent amount of recombinant FANCL (bottom). Anti-His western blots for detecting His-tagged FANCI and FANCD2 are shown. Anti-HA western blots of the same samples to detect HA-ubiquitin are shown in Figure S2C. (C) The amounts of FANCD2-Ub in the reactions shown in (B) were quantified and are plotted as a percentage of total FANCD2 (ubiquitinated FANCD2/total FANCD2). (D) Monoubiquitination of WT and nonubiquitinatable (lysine to arginine, KR) mutants of FANCI and FANCD2 by ∼100 nM core complex in the presence of DNA over 3 hr at 30°C. See also Figures S2 and S3.

Strikingly, the FA core complex is much more active than recombinant chicken FANCL (Figure 4). First, approximately five times more isolated FANCL is required to monoubiquitinate FANCD2 to the same level as FA core complex (Figure 4A, compare lanes 5 and 9; Figures S2A and S2B). Under these conditions, the FA core complex is also more specific, given that only isolated FANCL shows significant ubiquitin conjugation onto itself and UBE2T (Figure 4A, HA blot lanes 8 and 9). Second, when equimolar amounts of FA core complex and isolated FANCL are used in a time course of FANCD2 monoubiquitination, the rate of FANCD2 monoubiquitination is dramatically higher with FA core complex (Figures 4B, 4C, and S2C). Finally, FA core complex ubiquitinates FANCD2 on only the physiologically relevant lysine 563 (Figure 4D), whereas FANCL exhibits weak, but reproducible nonspecific, monoubiquitination of FANCD2^K563R^ (see Figure S3A, lanes 23 and 35) (Sato et al., 2012). Cumulatively, these data show that the FA core complex is much more active and more specific than isolated FANCL.

Purified FA core complex catalyzes only the physiologically relevant formation of monoubiquitin linkages because a single discrete ubiquitinated FANCD2 species is observed (Figure 3). In contrast, reactions containing isolated FANCL demonstrate weak poly- or multiple monoubiquitination activity, seen as higher-molecular-weight smears on the gel (Figure S2A, HA blot lanes 8 and 9). Also unlike the FA core complex (Figure 3B, lanes 2 and 5), recombinant FANCL has FANCD2 monoubiquitination activity even in the absence of FANCI (see Figure S3A, HA blot lane 3) (Alpi et al., 2008; Sato et al., 2012). Altogether, these data suggest that other FA core complex subunits most likely contribute to activity, substrate recognition, and specificity required to retain exclusive monoubiquitination activity on FANCD2 in the context of FANCI.

### UBE2T, but Not UBE2W, Functions as a Specific E2 for FANCD2 Monoubiquitination In Vivo and In Vitro

Although UBE2T is established as an E2 for FANCD2 monoubiquitination in vivo (Alpi et al., 2007, 2008; Machida et al., 2006), UBE2W also binds FANCL (Alpi et al., 2008; Zhang et al., 2011). We found that, in assays with FANCL, UBE2W weakly monoubiquitinates FANCD2 and FANCI, but this was not dependent on the presence of the FANCI-FANCD2 complex (Figure S3A, lanes 4, 6, 10, and 12), stimulated by DNA (Figure S3A, lanes 6 and 12), and not site specific (Figure S3A, lanes 26 and 38). Similar observations were made in assays with the FA core complex (Figure S4B). These data indicate that UBE2W mediates nonspecific monoubiquitination of the FANCI-FANCD2 complex in vitro. To investigate its in vivo role, we deleted *UBE2W* in DT40 cells and examined FA pathway activity (Figures S3C and S3D). Δ*UBE2W* cells did not display significant cellular sensitivity to MMC (Figure S3E) and had normal levels of basal and MMC-induced FANCD2 monoubiquitination, unlike Δ*UBE2T* or Δ*FANCB* cells (Figure S3F). Along with previous work (Alpi et al., 2007; Machida et al., 2006), our results emphatically show that UBE2W does not play a major role in site-specific monoubiquitination of FANCD2 and that UBE2T is the major E2 in the FA pathway.

### FANCI Is Not Ubiquitinated by a Purified FA Core Complex

FANCI monoubiquitination is presumed to be catalyzed by the FA core complex, but this modification is dispensable for DNA repair (Ishiai et al., 2008; Sims et al., 2007; Smogorzewska et al., 2007). We only observed weak FANCI monoubiquitination in our assay that was not affected by the presence of FANCD2 or DNA and did not significantly increase over time, suggesting that it may not be physiologically relevant (HA blots in Figures 3B, S1D, and S2). Therefore, the factors enhancing FANCI monoubiquitination remain elusive but could include an alternate chromatin and/or DNA context or interplay with ATR-dependent FANCI phosphorylation (Ishiai et al., 2008; Tomida et al., 2013).

### Purified FA Core Complex Lacking FANCC and FANCE Retains In Vitro Activity

**Figure 5:**
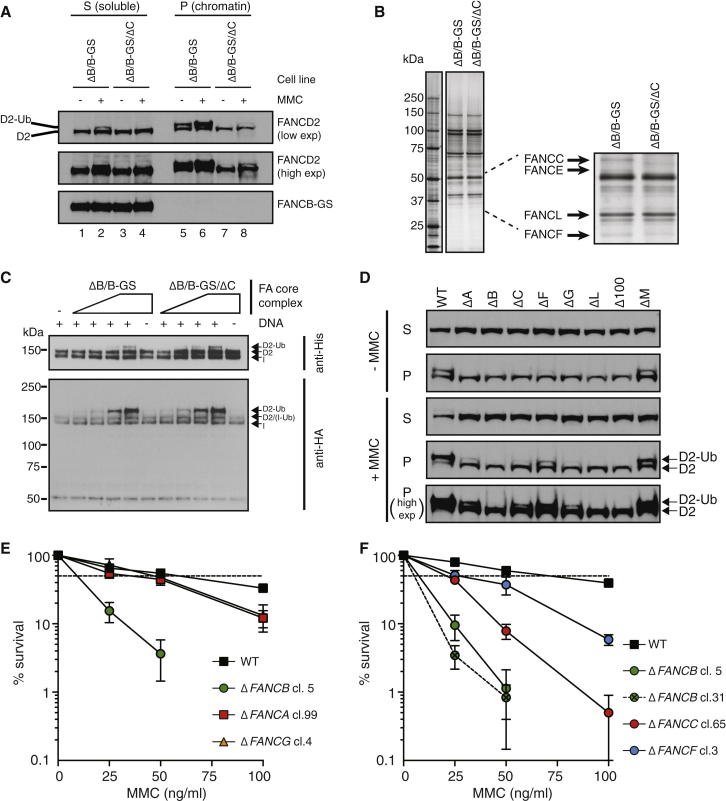
Deletion of *FANCC* Does Not Impact the In Vitro Ubiquitin Ligase Activity of the FA Core Complex, and the FANCB-FANCL-FAAP100 Subcomplex Is Essential for Monoubiquitination In Vivo (A) Western blots of Δ*FANCB/FANCB-GS* (ΔB/B-GS) and Δ*FANCB/FANCB-GS*/Δ*FANCC* (ΔB/B-GS/ΔC) cells (with and without MMC treatment) after subcellular fractionation into cytonucleoplasmic (S) and chromatin (P) fractions with anti-FANCD2. Low and high exposures are shown. (B) SDS-PAGE and silver stain analysis of the FA core complex purified from ΔB/B-GS and ΔB/B-GS/ΔC cell lines in parallel. The inset shows loss of FANCC and FANCE proteins and reduction of FANCF. (C) In vitro ubiquitination assays of the FA core complexes (∼80 nM) shown in (B) monitored by western blotting against FANCD2 and FANCI (anti-His) and ubiquitin (anti-HA). (D) DT40 cell lines with FA core complex subunit deletions were treated with MMC, fractionated into cytonucleoplasmic (S) and chromatin (P) fractions and subjected to western blotting with anti-FANCD2. An additional high exposure is shown for the bottom panel. (E and F) Cellular sensitivity of WT, Δ*FANCA*, Δ*FANCG*, Δ*FANCB*, Δ*FANCC*, and Δ*FANCF* DT40 cells after exposure to indicated doses of MMC. The mean percentage of survival ± SEM of three independent colony-formation assays are plotted relative to untreated cells. Δ*FANCB* cl. 31 is the unloxed parental line to Δ*FANCB* cl. 5. See also Figure S4.

FA can result from the loss of a single FA core complex subunit, which is thought to interfere with the integrity of the entire complex. To further investigate the stability and in vitro activity of the FA core complex, we deleted the *FANCC* gene (Niedzwiedz et al., 2004) in Δ*FANCB*/*FANCB-GS* cells. Cellular fractionation before and after *FANCC* deletion revealed that although FANCD2 monoubiquitination was significantly impaired, a residual amount was detectable in the chromatin-bound fraction after MMC treatment (Figure 5A, lane 8). This suggested that even with the loss of FANCC, the FA complex remains partially competent for FANCD2 monoubiquitination in vivo. To verify this, we purified the FA core complex from the Δ*FANCC*/Δ*FANCB*/*FANCB-GS* cell line. This yielded a preparation specifically lacking the deleted *FANCC* gene product and its known binding partner FANCE (Figure 5B) (Pace et al., 2002; Taniguchi and D’Andrea, 2002). FANCF, thought to bridge the FANCC-FANCE and FANCA-FANCG subunits (Léveillé et al., 2004), was also depleted. Thus, although *FANCC* deletion partially destabilized the complex through loss of FANCE and FANCF, the remaining subunits (FANCA, FANCB, FANCG, FANCL, and FAAP100) were stably associated and the yield of purified complex was similar to wild-type cells.

Surprisingly, when we assayed the FA core complex lacking FANCC and FANCE, FANCD2 monoubiquitination activity was comparable to the wild-type complex (Figure 5C). There was no discernable difference in the rate of monoubiquitination, the effect of DNA, or the lack of FANCI monoubiquitination. These data demonstrate that the FANCC-FANCE subcomplex is dispensable for the overall stability and catalytic activity of the FA core complex.

### Residual In Vivo FANCD2 Monoubiquitination Activity after Deletion of FANCA, FANCC, FANCF, or FANCG but Not FANCB, FANCL, or FAAP100

We were surprised that purified FA core complex lacking FANCC and FANCE (and with substantially reduced FANCF) had normal activity in vitro. To understand the roles of other core complex subunits, we took further advantage of the genetic tractability of the DT40 system. Specifically, we examined FANCD2 ubiquitination in vivo in a panel of DT40 cell lines in which each individual FA core complex subunit, with the exception of *FANCE*, had been deleted. Although very weak in comparison to wild-type or Δ*FANCM* cells, residual chromatin-associated FANCD2 monoubiquitination is still present in cells lacking FANCA, FANCC, FANCF, or FANCG (Figure 5D, bottom two panels). This is an unexpected result, given that work on human FA patient-derived lymphocytes shows an absence of FANCD2 monoubiquitination in all of the FA core complex complementation groups except FANCM (Garcia-Higuera et al., 2001; Singh et al., 2009).

In contrast, cells lacking FANCB, FANCL, or FAAP100 completely lost the ability to ubiquitinate FANCD2 (Figure 5D). This is consistent with FANCL’s role as the E3 ligase and the direct interaction of these three subunits (Ling et al., 2007). This surprising result demonstrates that, at least in DT40 cells, FANCB, FANCL, and FAAP100 are the only subunits whose deletion results in complete loss of FANCD2 monoubiquitination. To further establish that this striking finding is broadly applicable to the mammalian FA pathway, we examined FANCD2 monoubiquitination in mammalian cell lines (human, mouse, and hamster) with targeted disruption of a FA subunit and compared this to isogenic wild-type cell lines. Chromatin-bound FANCD2 from cells with targeted disruption of *FANCG*, but not *FANCB*, retains residual FANCD2 ubiquitination (Figure S4). Furthermore, residual FANCD2 monoubiquitination has recently been demonstrated in a panel of human cell lines with targeted disruption of FA core complex subunits with the exception of Δ*FANCB*, Δ*FANCL*, or Δ*FAAP100*, which have no monoubiquitination activity (Huang et al., 2014).

Ubiquitinated FANCD2 is thought to act as an adaptor for DNA repair enzymes, allowing incision at sites of DNA crosslinks and promoting repair by homologous recombination (Crossan and Patel, 2012). Therefore, residual FANCD2 ubiquitination in certain FA core complex subunit knockout cells (Figure 5D) would be expected to show attenuated cellular sensitivity to DNA damage in comparison to knockout cells with no FANCD2 ubiquitination. To test this, we exposed DT40 cells to MMC and determined their survival. As shown in Figures 5E and 5F, Δ*FANCB* cells are more sensitive to MMC than Δ*FANCA*, Δ*FANCG*, Δ*FANCC*, and Δ*FANCF* cells. Thus, the amount of FANCD2 monoubiquitination correlates with MMC sensitivity in cells.

### Recombinant B-L-100 Monoubiquitinates FANCD2

**Figure 6:**
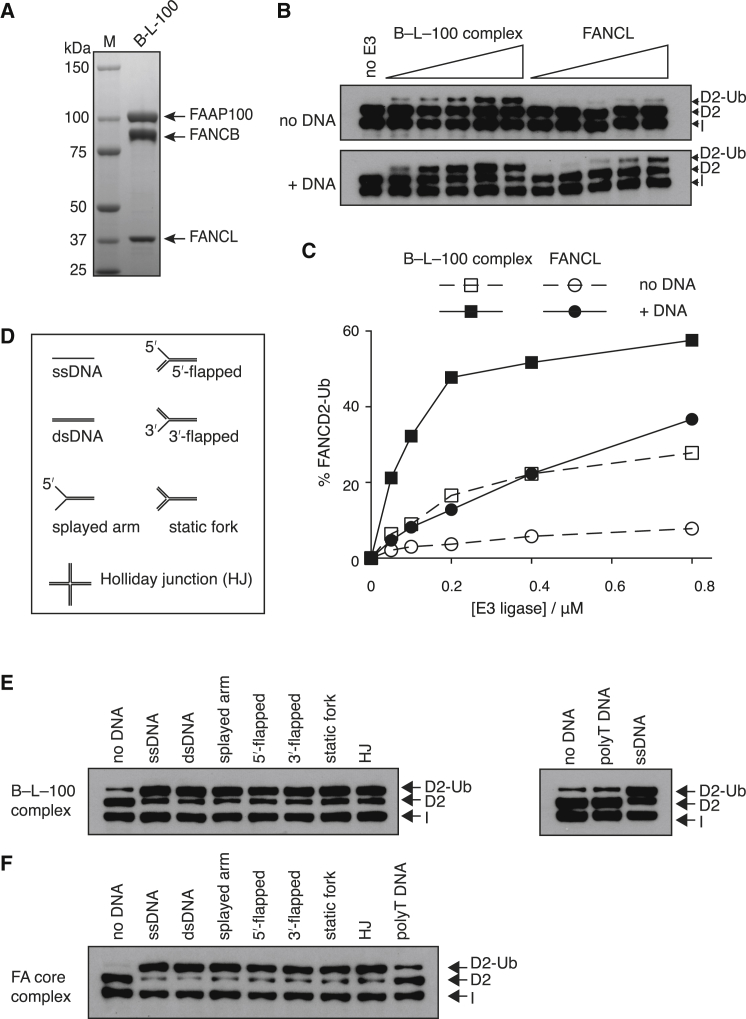
In Vitro FANCD2 Monoubiquitination by the B-L-100 Subcomplex (A) Coomassie Blue SDS-PAGE analysis of purified chicken recombinant B-L-100. (B) FANCD2 monoubiquitination by the B-L-100 subcomplex and isolated FANCL in 2-fold dilution series starting at 0.8 μM in the absence and presence of DNA analyzed by western blotting with anti-His. (C) The amounts of FANCD2-Ub in the reactions shown in (B) were quantified and plotted as a percentage of total FANCD2 (ubiquitinated FANCD2/ total FANCD2). (D) Schematic of different DNA substrates. ss, single-stranded; ds, double-stranded. (E and F) FANCD2 monoubiquitination by the B-L-100 subcomplex (E) or FA core complex (F) in the presence of 1 μM DNA substrates indicated in (D). Reactions were performed for 90 min with 200 nM B-L-100 or FA core complex. Right, a comparison between 49-mer ssDNA and polyT DNA. See also Figure S5.

FANCC and FANCE are biochemically dispensable for FANCD2 ubiquitination by a purified FA core complex (Figure 5C), and only FANCB, FANCL, and FAAP100 are genetically essential in vivo for monoubiquitination (Figure 5D). Therefore, we reasoned that FANCB, FANCL, and FAAP100 could constitute the catalytic core of the FA core complex. We expressed and purified a complex of B-L-100 (Figure 6A). In vitro reconstitution assays show that B-L-100 is more active than FANCL alone (Figures 6B and 6C). In the presence of DNA, the B-L-100 subcomplex is 5- to 6-fold more active at ubiquitinating FANCD2 than isolated FANCL and thus comparable with the intact core complex. However, unlike the intact core complex, weak FANCD2 ubiquitination in the absence of FANCI (Figure S5A) and very weak multiple ubiquitination could be observed (Figure S5B).

Interestingly, the B-L-100 subcomplex retained significant activity in the absence of DNA (compare Figures 4C and 6C). To gain insight into the mechanism of DNA stimulation, we tested a panel of DNAs with different structures for their abilities to stimulate FANCD2 monoubiquitination. A variety of different DNA substrates stimulated the reaction (Figures 6D–6F and S5C). Intriguingly, unstructured polyT DNA did not stimulate the activity of B-L-100 or the intact core complex to the same extent as other DNAs (Figures 6E and 6F). In comparison to B-L-100, the intact core complex has lower basal FANCD2 monoubiquitination activity in the absence of DNA. This suggests DNA may be required to activate the FA core complex.

### USP1 Deletion Permits Accumulation of FANCD2 Monoubiquitination in Cells with Residual Activity

**Figure 7:**
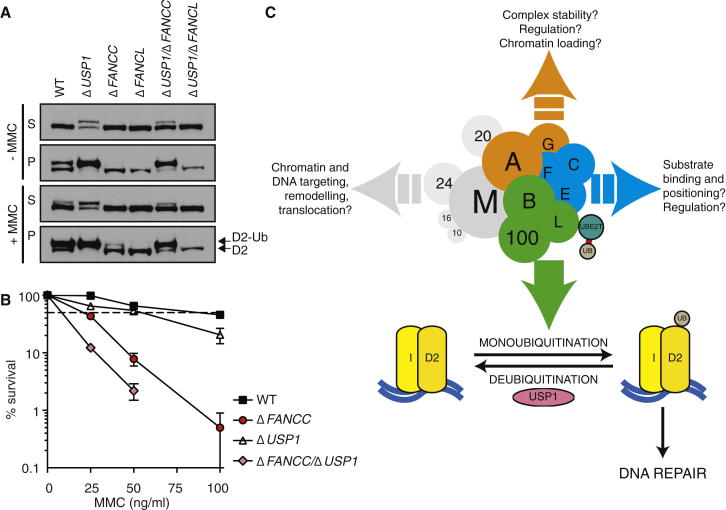
*USP1* Deletion Restores FANCD2 Monoubiquitination but Not Sensitivity to MMC in Δ*FANCC* Cells (A) DT40 cell lines with indicated genotypes were treated with MMC, fractionated into cytonucleoplasmic (S) and chromatin (P) fractions, and analyzed by western blotting with anti-FANCD2. (B) Cellular sensitivity of DT40 cells after exposure to indicated doses of MMC. The mean percentage of survival ± SEM of three independent colony-formation assays are plotted relative to untreated cells. (C) The FA core complex is comprised of multiple modules. B-L-100 (green) is central to the monoubiquitination step. The A-G (orange) and C-E-F (blue) modules may affect this catalytic function through regulatory or stabilizing roles. They may also serve distinct functions independent of ubiquitination in FA DNA repair. Subunits shown to be dispensable for ubiquitination in vivo are in gray. See also Figure S6.

The deubiquitinase enzyme USP1, in complex with UAF1, has been shown to deubiquitinate FANCD2 (Nijman et al., 2005). USP1 disruption leads to constitutive, chromatin-targeted monoubiquitinated FANCD2 (Kim et al., 2009; Oestergaard et al., 2007). To understand the functional importance of weak residual FANCD2 ubiquitination found in a FA-core-complex-defective cell line, we targeted both *USP1* and *FANCC* in DT40 cells (Figure S6A). Strikingly, FANCD2 monoubiquitination was restored in the double mutant, supporting the observation of a partially active core complex in Δ*FANCC* cells (Figures 7A, S6B, S6D, and S6E). However, ubiquitination was not induced upon DNA damage, and monoubiquitinated FANCD2 was largely chromatin bound, though it was not as heavily enriched on chromatin as in Δ*USP1* cells (Oestergaard et al., 2007). Another target of USP1 activity, PCNA, was not affected in the double mutant (Figures S6B and S6C). As previously reported (Oestergaard et al., 2007), a Δ*USP1*/Δ*FANCL* mutant showed no rescue of FANCD2 ubiquitination, given that the catalytic activity of the core complex was fully compromised (Figure 7A).

Although monoubiquitinated FANCD2 was present in Δ*USP1/*Δ*FANCC* cells, the double mutant was more sensitive to MMC than either single mutant (Figure 7B). In comparison, we have previously shown that Δ*USP1*/Δ*FANCL* cells are not more sensitive to DNA damage than the *FANCL* mutant alone (Oestergaard et al., 2007). Altogether, these results support the notion of epistasis between the gene products essential for monoubiquitination (FANCB, FANCL, and FAAP100) and deubiquitination (USP1) events in crosslink repair. Even though the residual FANCD2 ubiquitination in Δ*FANCC* cells is sufficient to partially attenuate MMC sensitivity in comparison to Δ*FANCB* cells that entirely lack ubiquitination (Figure 5F), simply restoring the ubiquitinated FANCD2 (by deleting Δ*USP1*) in this context is not sufficient to restore a functional FA DNA repair response.

## Discussion

In this study, we have combined a stringent biochemical purification strategy with genetic analyses in DT40 cells to establish a system for mechanistic interrogation of the FA pathway. We report the purification of a native FA core complex and the reconstitution of its FANCD2 monoubiquitination activity in vitro. The intact complex is more active and specific than the FANCL subunit alone. Within the intact complex, we identify a minimal subcomplex of just three subunits (B-L-100) that constitutes the essential machinery required for robust FANCD2 ubiquitination (Figure 7C). Understanding how FANCB and FAAP100 enhance the activity of FANCL in this subcomplex will be an important direction for future study. They may stabilize the FANCL protein or support a conformation permissive for binding to both UBE2T and FANCD2 in order to enhance substrate recognition, positioning, and modification.

Surprisingly, even though mutation in any subunit was thought to destabilize and inactivate the FA core complex in patients, not all subunits are required for monoubiquitination. We verified the catalytic competence of FA core complex lacking FANCC with both in vivo (Figures 5A, 5D, and 7A) and in vitro (Figure 5C) methods. The reduced level of in vivo ubiquitination in Δ*FANCC* cells may result from inefficient regulation, subcellular localization, or unproductive substrate binding (Gordon et al., 2005; Pace et al., 2002). It is possible that FANCC and other core complex subunits directly regulate the monoubiquitination reaction (positively or negatively), help recruit and/or position substrates, or indirectly control the catalytic activity in vivo through stability and/or localization of the FA core complex. Alternatively, given that a mutation in any single core complex component (not just *FANCB*, *FANCL*, or *FAAP100*) manifests in FA, the core complex or its subcomplexes could have additional roles in DNA repair independent of ubiquitination (Matsushita et al., 2005). Nevertheless, our work provides clear evidence that the B-L-100 subcomplex is the monoubiquitination module because DT40 cells that have a deletion of any other subunit retain residual FANCD2 monoubiquitination. To date, residual FANCD2 monoubiquitination has not been detected in FA patient-derived cell lines, although it is observed in gene-targeted mammalian cells (Figure S4).

A number of different DNA structures stimulate the in vitro ubiquitination of FANCD2. This is not unexpected for two reasons. First, the FA pathway is activated in vivo by a diverse range of DNA-damaging and replication-stress-inducing agents that are likely to elicit different DNA conformations, structures, and adducts (Garcia-Higuera et al., 2001; Howlett et al., 2005). Second, studies in *Xenopus* egg extracts reveal that an array of exogenous DNA substrates can induce FANCI/FANCD2 monoubiquitination (Räschle et al., 2008; Sareen et al., 2012; Sobeck et al., 2007). Given that unstructured polyT had a reduced effect, structured and/or duplex DNA may be required to promote a conformational change that makes the K563 acceptor lysine in FANCD2 accessible. This may be mediated by FANCI, which is known to bind FANCD2 and structured DNA substrates (Joo et al., 2011; Longerich et al., 2009; Sato et al., 2012; Yuan et al., 2009).

Although our purification shows that the intact complex exists and can be purified, independent of induced DNA damage (Figure 2B) (Alpi et al., 2007), the subunits were present in nonuniform amounts (Table S1) and could be grouped based on their relative abundances: B-L-100, C-E-F, and A-G (Figure 7C). The existence of FA subcomplexes is consistent with earlier work (Garcia-Higuera et al., 2001, 2000; Ling et al., 2007; Medhurst et al., 2006; Thomashevski et al., 2004) and could represent different stoichiometries within the assembled complex, assembly intermediates, and/or functional modules. For example, although FANCL has been shown to directly bind FANCD2 and FANCI (Cole et al., 2010; Hodson et al., 2011; Seki et al., 2007), C-E-F could represent a functional module that regulates these substrate interactions via FANCE-FANCD2 associations or impinges upon substrate accessibility by USP1 (Gordon et al., 2005; Nookala et al., 2007; Pace et al., 2002; Polito et al., 2014).

FANCF could act as a scaffold to anchor both the A-G module (Léveillé et al., 2004) and the FANCM-FAAP24-FAAP10-FAAP16 module implicated in chromatin targeting, suppression of crossover recombination, and checkpoint signaling (Collis et al., 2008; Deans and West, 2009; Rosado et al., 2009). The A-G module may also function in chromatin targeting of the complex suggested by data showing intrinsic DNA binding of FANCA (Yuan et al., 2012) and the association of FANCA with FAAP20, a ubiquitin-binding-zinc-finger-containing protein that binds ubiquitin chains assembled by RNF8 and promotes chromatin targeting of the core complex and FANCD2 in vivo (Ali et al., 2012; Leung et al., 2012; Yan et al., 2012).

Our finding that combined *USP1* and *FANCC* deletion restores monoubiquitinated FANCD2 but cannot rescue MMC sensitivity challenges the broadly held view of a linear FA pathway in which substrate ubiquitination by the FA core complex alone leads to DNA repair. The ubiquitinated FANCD2 in Δ*USP1*/Δ*FANCC* cells, although near wild-type levels, is substantially chromatin bound and not induced by DNA damage. Clearly, the ubiquitination and deubiquitination of FANCD2 must be a tightly regulated response in a functional FA pathway. That Δ*USP1*/Δ*FANCC* cells are more sensitive than either single mutant suggest that the FA core complex may serve other functions in DNA repair in addition to ubiquitination. Elucidating these functions may ultimately explain why the FA core complex has such a highly conserved yet poorly understood macromolecular composition.

Our work establishes a comprehensive genetic, biochemical, and structural framework to dissect the mechanism of monoubiquitination by the FA core complex. Using this system, it may now be possible to interrogate the activity of the complex from different physiological states, different subcellular locations, in response to cell-cycle progression and, critically, in response to DNA damage when the FA pathway is activated.

## Experimental Procedures

Detailed purification protocols and generation of cell lines are described in the Supplemental Information. The tandem affinity purification scheme is modified from Bürckstümmer et al. (2006) and was performed at 4°C. In brief, to purify a FA core complex, ΔB/B-GS, or ΔB/B-GS/ΔC DT40 cells were lysed in GS buffer (50 mM HEPES [pH 8.0], 150 mM NaCl, 5% glycerol, 0.1% Igepal CA-630, 1.5 mM MgCl_2_, 25 mM NaF, 2 mM Na_3_VO_4_, 40 mM β-glycerophosphate, 1 mM phenylmethylsulfonyl fluoride, 10 mM β-mercaptoethanol [BME], and PhosSTOP inhibitor cocktail [Roche] and protease inhibitor cocktail) by passing through 19 G and 25 G needles multiple times. After clarification, the lysate was incubated with IgG-agarose beads (Sigma-Aldrich) for 2 hr with gentle rotation. The beads were washed with wash buffer (50 mM HEPES [pH 8.0], 150 mM NaCl, 10% glycerol, and phosphatase/protease inhibitors) and then wash buffer supplemented with 5 mM BME. To elute, the beads were incubated with TEV protease at 16°C for 2 hr with gentle agitation. For the second affinity step, the supernatant was incubated with Ultralink Streptavidin Plus Resin (Pierce) with gentle rotation for 1 hr, washed with 100 bed volumes of wash buffer supplemented with 5 mM BME, and eluted with wash buffer supplemented with 3 mM BME and 6 mM D-biotin (Sigma-Aldrich). The complex was resolved by SDS-PAGE and analyzed by Coomassie, silver (Sigma-Aldrich), or SYPRO-Ruby (Lonza) staining. Bands were excised, digested, and identified by tandem mass spectrometry (data not shown) with multiple independent purifications. The major species identified in each band is indicated in Figure 2B. Purified complex was used fresh, immediately after purification, for downstream biochemical analyses. A titration against known concentrations of recombinant FANCL was used to approximate the concentration of prepared FA core complex (normalized to its FANCL band) on SDS-PAGE. We estimate that, from a ∼3 g DT40 cell pellet (2–4 L cell culture), we purified ∼5 μg FA core complex.

Western blotting was performed after SDS-PAGE on 4%–12% Bis-Tris gels or 3%–8% Tris-Acetate gels (Life Technologies) and transfer to polyvinylidene fluoride membranes (Millipore). The antibodies used were HRP-conjugated anti-HA (F-7, Santa Cruz Biotechnology), HRP-conjugated anti-His (H-3, SCBT), anti-β-actin (AC-15, Sigma-Aldrich), anti-chicken FANCG (Alpi et al., 2007), anti-chicken FANCM (Mosedale et al., 2005), or anti-chicken FANCD2 (gift of Michael Hodskinson, Medical Research Council Laboratory of Molecular Biology).

For ubiquitination assays, reactions in 10–20 μl volumes were performed in a buffer comprising 50 mM HEPES (pH 7.5), 64 mM NaCl, 4% glycerol, 5 mM MgCl_2_, 2 mM ATP, and 0.5 mM dithiothreitol. In all reactions, 75 nM E1 (Boston Biochem) and 1 μM E2 (UBE2T or UBE2W) enzymes, 1 μM substrate (FANCI, FANCI^K525R^, FANCD2, and FANCD2^K563R^), 50 μM 5′-flapped DNA (nucleotide concentration, unless otherwise stated), and 20 μM HA-ubiquitin (Boston Biochem) were used unless otherwise stated. E3 (FANCL or FA core complex) concentrations are indicated in the figures. Optimal E2 and FANCL concentrations were determined by titrations. All reactions were incubated at 30°C for 90 min (or for indicated times as part of a time course) and quenched by the addition of LDS sample buffer (Life Technologies). Samples were analyzed by western blotting.

Additional details of experimental procedures are contained in the Supplemental Information.

## Author Contributions

E.R., K.J.P., and L.A.P. designed the study, interpreted data, and wrote the paper. E.R. planned and executed the majority of experiments. E.R., V.H.O., F.L., and M.W. generated cell lines. F.L. performed clonogenic assays. G.L.D. generated baculoviruses. L.A.P. and K.J.P. supervised the project. V.H.O. and F.L. contributed equally to this work.

## Supplemental Information

Document S1. Supplemental Experimental Procedures and Figures S1–S7, and Table S1

Document S2. Article plus Supplemental Information
