# Supplementary material for: The Genetic and Biochemical Basis of FANCD2 Monoubiquitination
Source: Mol Cell. 2014 Jun 5;54(5):858–69. doi: 10.1016/j.molcel.2014.05.001 (PMC4051986; doi:10.1016/j.molcel.2014.05.001)
Supplement: Document S1. Supplemental Experimental Procedures and Figures S1–S7, and Table S1 [file mmc1.pdf]

Molecular Cell, Volume *54*

Supplemental Information

The Genetic and Biochemical Basis of FANCD2 Monoubiquitination

Eeson Rajendra, Vibe H. Oestergaard, Frédéric Langevin, Meng Wang, Gillian L. Dornan, Ketan J. Patel, and Lori A. Passmore

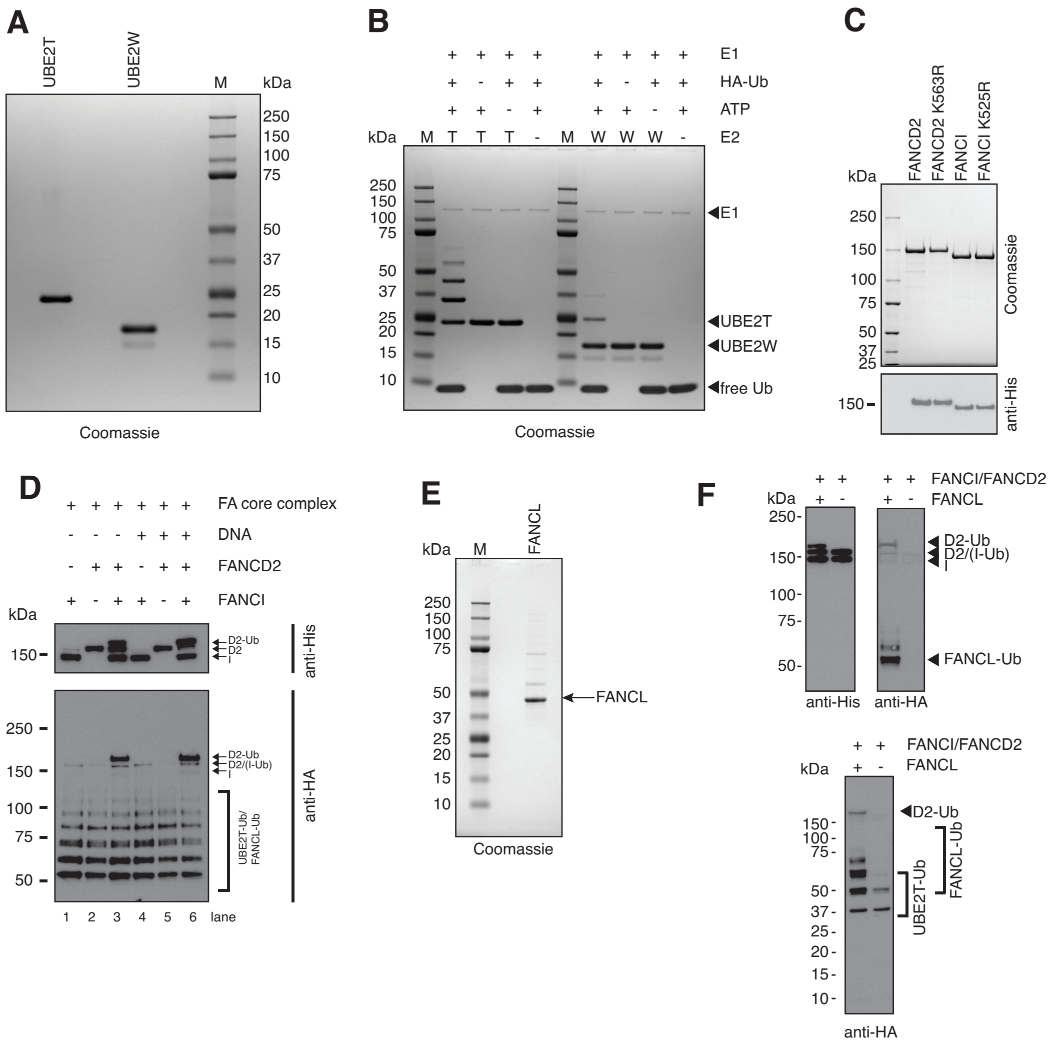

Figure S1, related to Figure 3. Purification of components and validation of the FANCD2 ubiquitination assay. (**A**) Coomassie blue-stained SDS-PAGE of purified UBE2T and UBE2W. (**B**) To check the activities of UBE2T and UBE2W, autoubiquitination assays were performed in the presence of 75 nM E1, 20 μM HA-ubiquitin, 2 mM ATP and 5 μM E2 enzyme (UBE2T, T or UBE2W, W). Assays were analyzed by SDS-PAGE followed by Coomassie blue staining. M is molecular weight marker. (**C**) Coomassie blue-stained SDS-PAGE gel (top) and Western blot using anti-His antibody (bottom) of purified chicken His-tagged FANCD2, FANCI and non-ubiquitinatable mutants in which the target acceptor lysines are mutated to arginines (FANCD2^{K563R} and FANCI^{K525R}). (**D**) *In vitro* reconstitution of monoubiquitination of FANCI, FANCD2 and the FANCI-FANCD2 complex in the absence or presence of 5′-flapped DNA after an extended incubation of the reactions presented in Figure 3B (90 min at 30 °C followed by 12 h at room temperature). (**E**) Coomassie blue-stained SDS-PAGE of purified chicken FANCL. (**F**) Reconstitution of FANCL-mediated FANCD2 monoubiquitination in the presence of FANCI. Reactions were resolved by 3-8% (upper panels) and 4-12% (bottom panel) SDS-PAGE and analysed by Western blotting with antibodies against FANCI-FANCD2 (anti-His) or ubiquitin (anti-HA). Unmodified FANCI and FANCD2 showed mild cross-reactivity with the anti-HA antibody.

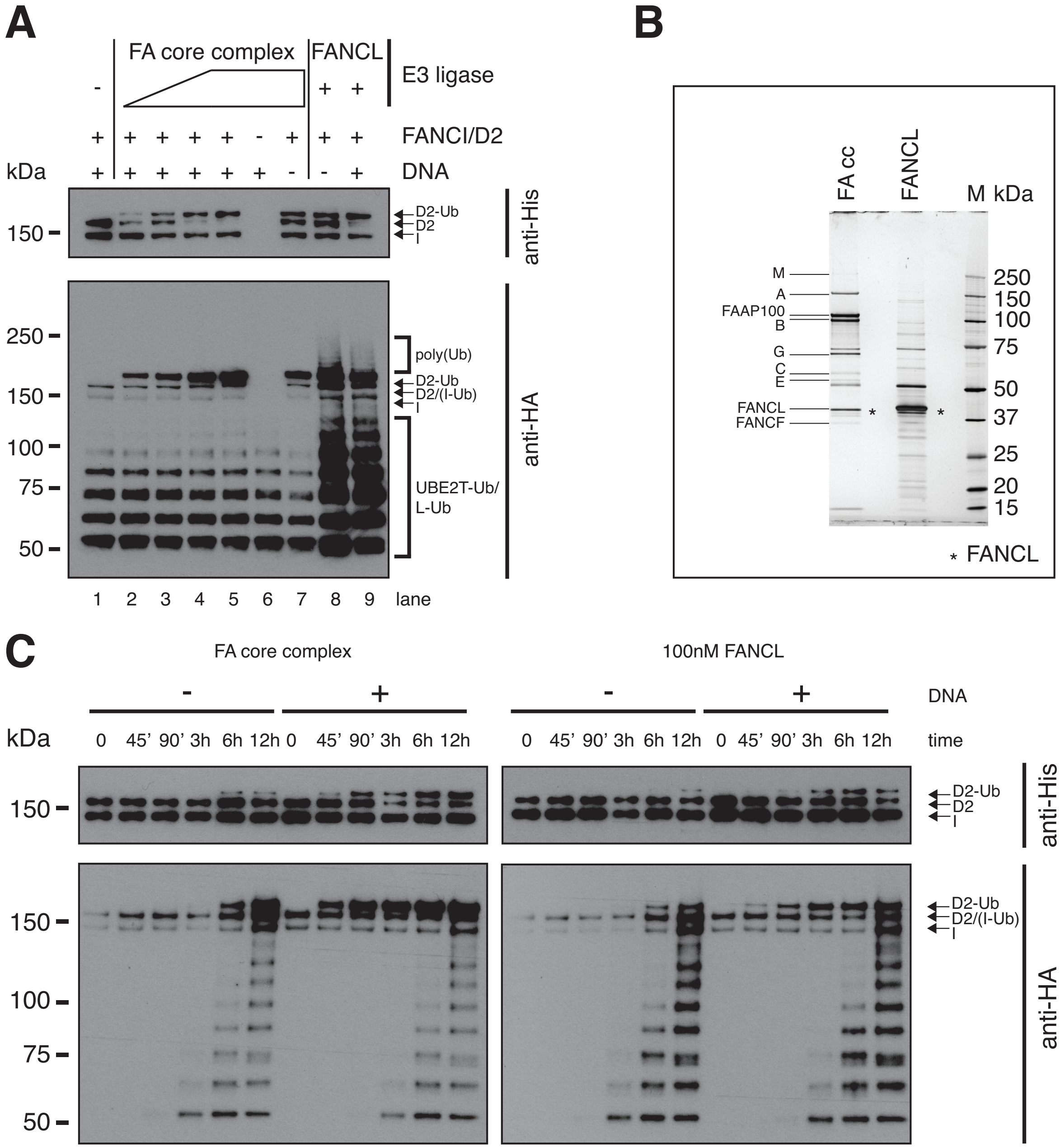

Figure S2, related to Figure 4. The FA core complex has higher activity than FANCL alone. (**A**) Titration of the FA core complex in a two-fold dilution series is compared to isolated FANCL in the monoubiquitination assay after an extended incubation (90 min at 30 °C followed by 12 h at room temperature) of the reactions presented in Figure 4A. (**B**) Silver-stained gel showing relative amounts of the purified FA core complex (at the highest concentration, lanes 5-7 of Figure 4A and S2A) and isolated FANCL used in the reactions presented in Figure 4A and S2A. Residual linker peptide (after GST cleavage) causes slower migration of recombinant FANCL compared to endogenous FANCL (indicated by asterisks). (**C**) Time course of FANCD2 monoubiquitination by the intact FA core complex (left) and a molar equivalent amount of recombinant FANCL (right). Anti-His blots detect His-tagged FANCD2 and FANCI (reproduced from Figure 4B); anti-HA blots detect HA-ubiquitin.

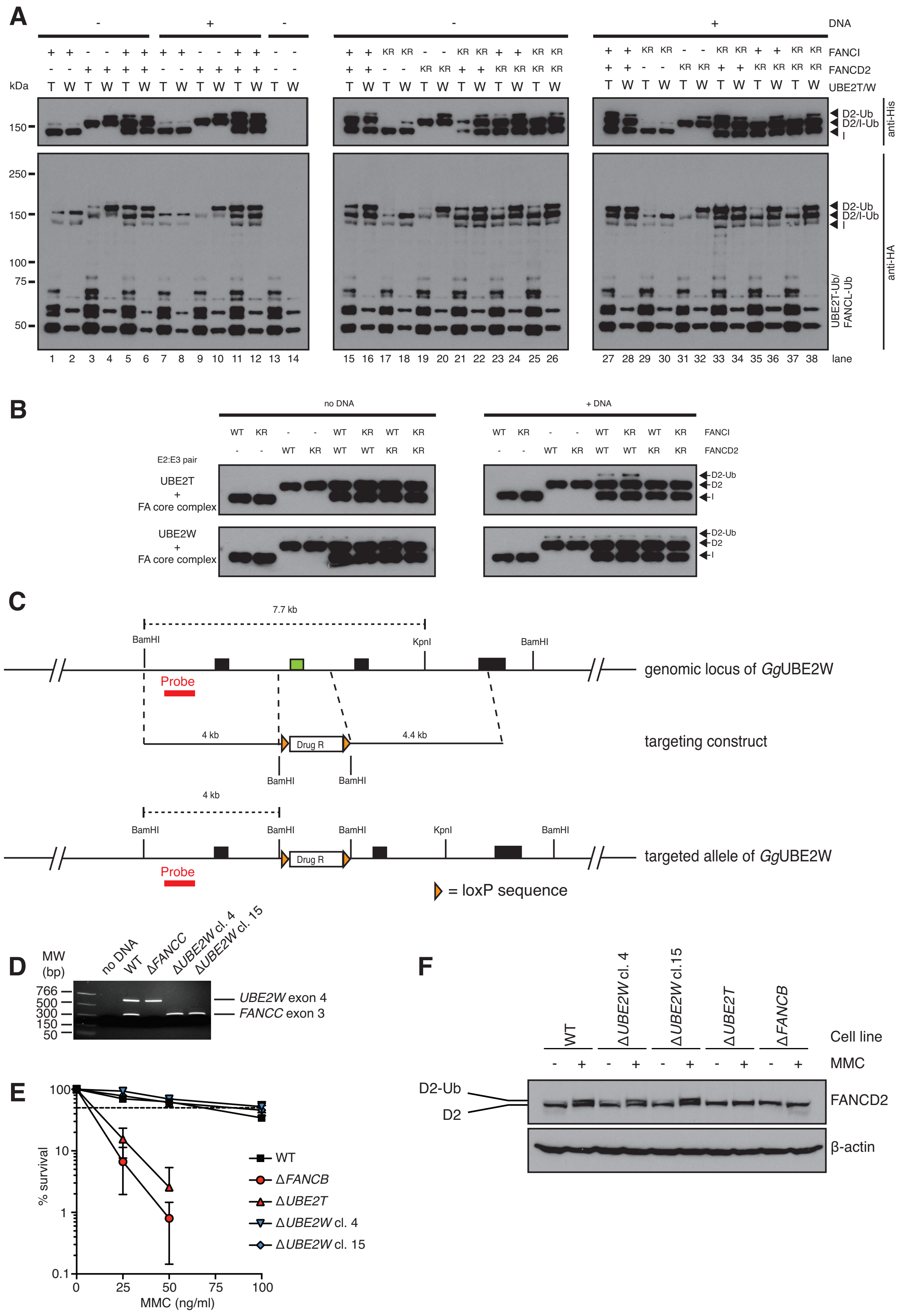

A
DNA
FANCI
FANCD2
UBE2T/W
kDa
250
150
100
75
50
D2-Ub
D2/I-Ub
I
anti-His
anti-HA
UBE2T-Ub/
FANCL-Ub
lane
B
no DNA
+ DNA
E2:E3 pair
UBE2T
+
FA core complex
UBE2W
+
FA core complex
D2-Ub
D2
I
C
7.7 kb
BamHI
KpnI
Probe
genomic locus of GgUBE2W
4 kb
4.4 kb
Drug R
targeting construct
targeted allele of GgUBE2W
= loxP sequence
D
MW
(bp)
766
500
300
150
50
no DNA
WT
ΔFANCC
ΔUBE2W cl. 4
ΔUBE2W cl. 15
UBE2W exon 4
FANCC exon 3
E
% survival
MMC (ng/ml)
WT
ΔFANCB
ΔUBE2T
ΔUBE2W cl. 4
ΔUBE2W cl. 15
F
WT
ΔUBE2W cl. 4
ΔUBE2W cl.15
ΔUBE2T
ΔFANCB
Cell line
MMC
D2-Ub
D2
FANCD2
β-actin

Figure S3, related to Figure 4. Ubiquitination of FANCI and FANCD2 in the presence of UBE2T and UBE2W. (**A**) Ubiquitination of wild-type and non-ubiquitinatable (KR) FANCI and FANCD2, in the absence and presence of DNA was reconstituted with recombinant FANCL (1 μM). Assays were performed with either UBE2T (T) or UBE2W (W), both of which have been shown to bind FANCL. Reactions were analysed by Western blotting with antibodies against FANCI-FANCD2 (anti-His) or ubiquitin (anti-HA). Nonspecific monoubiquitination by UBE2W (odd lanes, W) is in agreement with recent reports showing that UBE2W can mediate *in vitro* monoubiquitination of the amino groups of substrate N-termini, not a specific internal acceptor lysine (Scaglione et al., 2013; Tatham et al., 2013).
(**B**) Ubiquitination of wild-type (WT) and non-ubiquitinatable (KR) FANCI and FANCD2, in the absence and presence of DNA was reconstituted with the FA core complex (~ 65 nM) with either UBE2T or UBE2W for 90 min. Western blots with anti-His antibody are shown.
(**C**) Targeting strategy for the generation of the Δ*UBE2W* DT40 cell line. Exons are shown as green (exon 4) or black boxes. (**D**) PCR confirmation of genomic targeting of *UBE2W* and *FANCC* using oligos to amplify the deleted exons. (**E**) Cellular sensitivity of WT, Δ*UBE2T*, Δ*FANCB* and two independently generated clones of Δ*UBE2W* DT40 cells after exposure to indicated doses of mitomycin C (MMC). The mean % survival +/– S.E.M. of three independent experiments are plotted, relative to untreated cells. The dotted line indicates 50% survival showing that Δ*UBE2T* and Δ*FANCB* cell lines have approximately 20-fold lower LD50s than WT or Δ*UBE2W* cells. (**F**) Basal and MMC-induced FANCD2 monoubiquitination were examined in cell extracts of WT, Δ*UBE2W*, Δ*UBE2T* and Δ*FANCB* DT40 cells lines by Western blotting. FANCD2 ubiquitination is intact in Δ*UBE2W* cells.

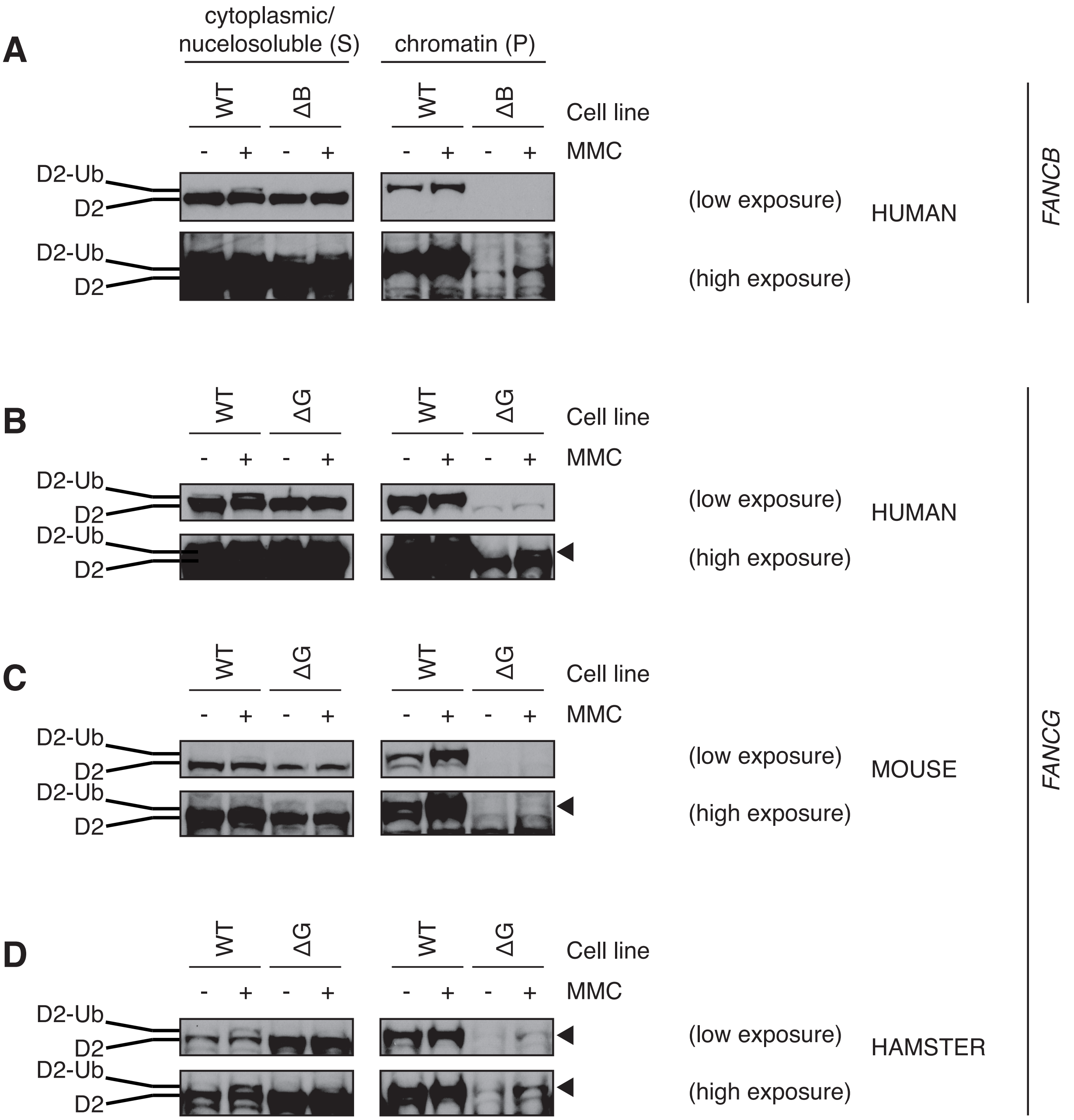

Figure S4, related to Figure 5. Residual FANCD2 monoubiquitination is observed in mammalian cells. Isogenic pairs of mammalian cell lines with targeted deletions of indicated FA core complex subunit deletions were treated with mitomycin C (MMC), fractionated into cytonucleoplasmic (S) and chromatin (P) fractions, and analyzed by Western blotting with an anti-FANCD2 antibody. In agreement with results in chicken DT40 cells, FANCD2 ubiquitination was (**A**) ablated in Δ*FANCB* human NALM6 cells (Nomura et al., 2007) but residual ubiquitination could be observed in Δ*FANCG* cells from (**B**) human (RKO cells; Gallmeier et al., 2006), (**C**) mouse (Koomen, 2002) and (**D**) hamster (NM3 CHO cells; Wilson et al., 2001). Residual ubiquitination is indicated by arrowheads.

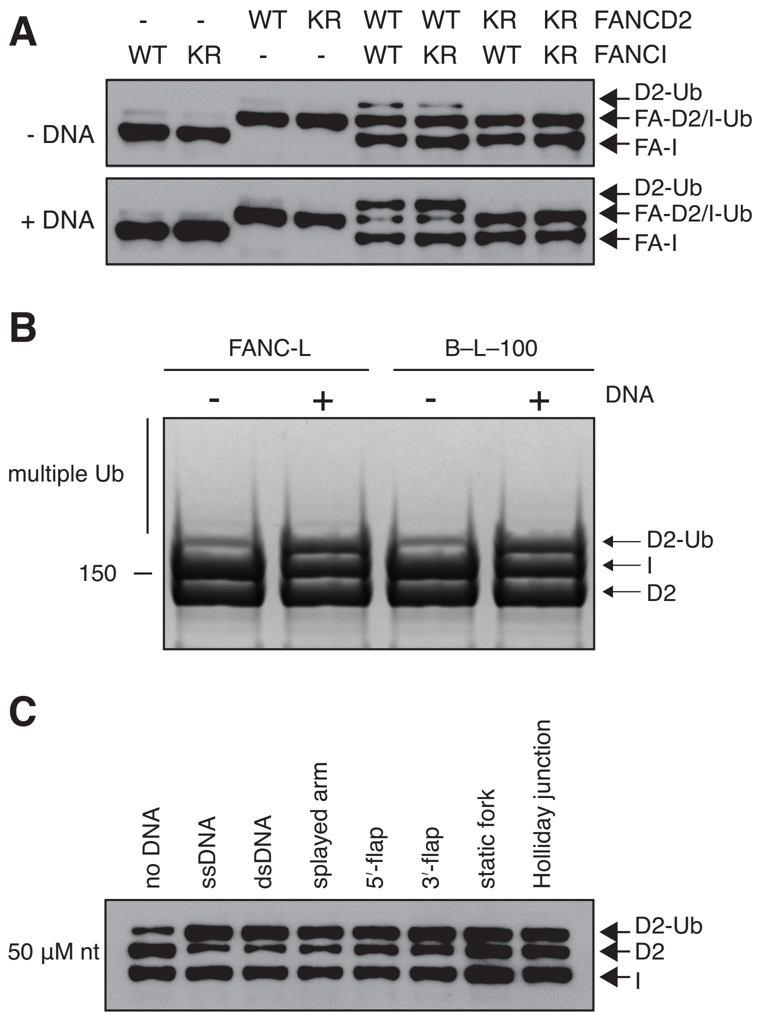

Figure S5, related to Figure 6. FANCD2 monoubiqutination by the FANCB–FANCL–FAAP100 (B–L–100) subcomplex. (**A**) Monoubiquitination of wild-type and non-ubiquitinatable (lysine to arginine, KR) mutants of FANCI and FANCD2 in the absence and presence of DNA by 200 nM B–L–100 for 90 min at 30 °C. Wild-type FANCD2 is ubiquitinated in the presence of FANCI or $FANCI^{K525R}$. Isolated FANCI and FANCD2 (WT and KR) are also weakly modified. (**B**) Coomassie blue-stained SDS-PAGE gel of FANCD2 ubiquitination by 0.8 μM FANCL and 0.15 μM B–L–100. These E3 concentrations allow similar amounts of FANCD2 monoubiquitination (see Figure 6C). FANCL shows increased multi-ubiquitination events on FANCD2. (**C**) Stimulation of FANCD2 monoubiquitination by 200 nM B–L–100 in the presence of different DNA substrates at 50 μM total nucleotide concentration (compared to 1uM of the DNA molecules, equimolar to the FANCI-FANCD2 substrates, in Figure 6E).

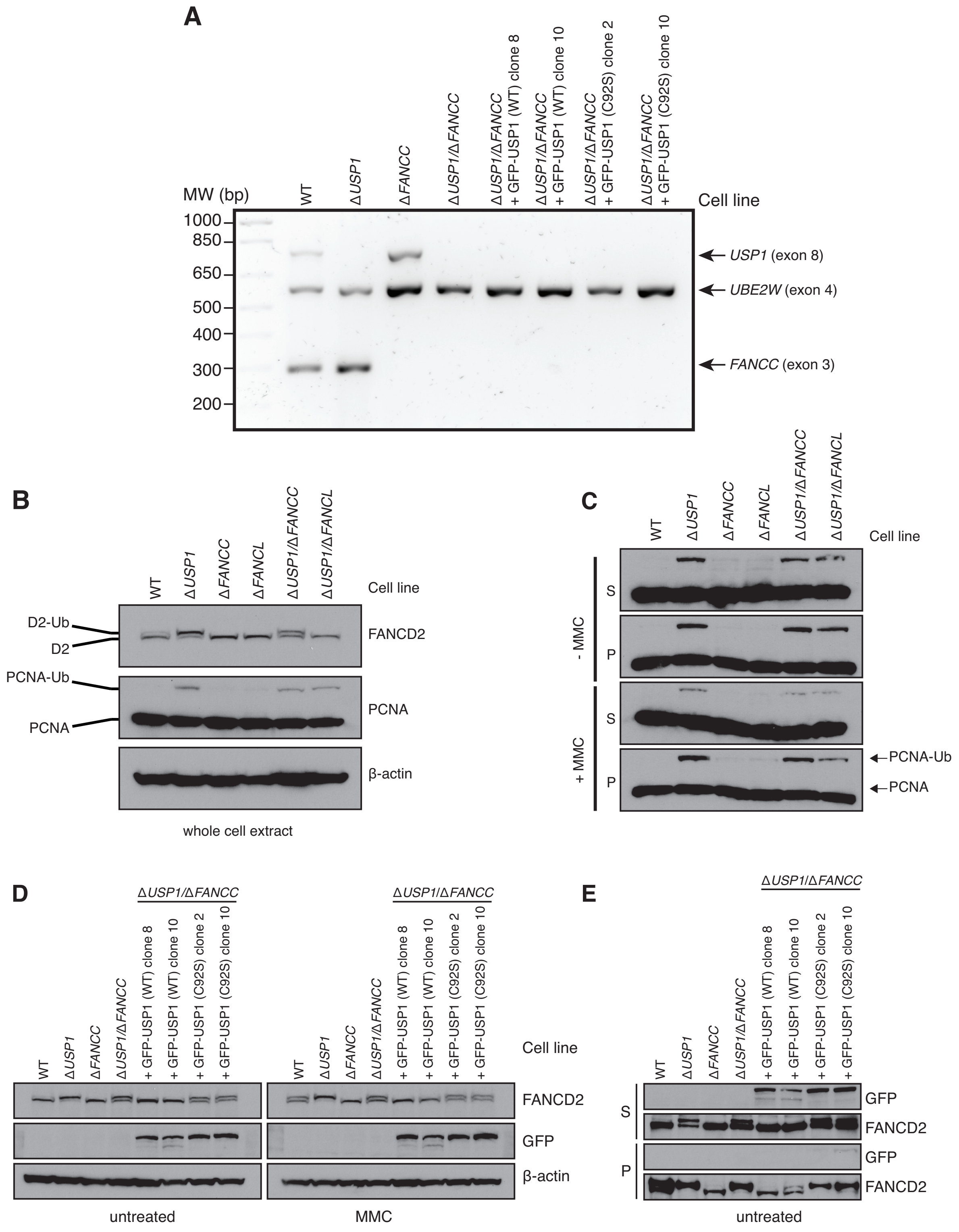

Figure S6, related to Figure 7. Genetic analysis of FANCD2 ubiquitination in Δ*USP1*/Δ*FANCC* DT40 cells. (**A**) PCR confirmation of genomic targeting of DT40 cells with indicated genotypes using oligos to amplify a region of *UBE2W* and the deleted regions of *USP1* and *FANCC*. (**B**) Western blots of whole cell extracts using anti-FANCD2, anti-PCNA

and anti-ß-actin antibodies. PCNA monoubiquitination is enhanced in Δ*USP1* cells but is not affected by additional loss of *FANCC* or *FANCL*. (**C**) Anti-PCNA Western blot of cyto-nucleosoluble (S) and chromatin (P) fractions in the presence and absence of mitomycin C (MMC) treatment. (**D**) Western blot of FANCD2 monoubiquitination in whole cell extracts in the absence or presence of MMC. Stable expression of GFP-USP1 (WT), but not a catalytically inactive mutant GFP-USP1 (C92S; Oestergaard et al., 2007), restores FANCD2 de-ubiquitination in Δ*USP1*/Δ*FANCC* cells. (**E**) Western blot of FANCD2 monoubiquitination in cytonucleosoluble (S) and chromatin (P) fractions. Deubiquitination is restored by stable expression of GFP-USP1 (WT) in Δ*USP1*/Δ*FANCC* cells. Expression level of GFP-USP1 (WT) correlates with degree of rescue (compare clones 8 and 10).

FA protein	Relative abundance
A	0.25 +/- 0.01
B	1.00
C	0.09 +/- 0.02
E	0.08 +/- 0.02
F	0.09 +/- 0.01
FAAP100	1.15 +/- 0.06
G	0.30 +/- 0.04
L	1.03 +/- 0.15
M	0.02 +/- 0.01

Table S1, related to Figure 2. Quantitation of the relative abundances of FA core complex subunits. Purified FA core complex was analyzed by SDS-PAGE followed by SYPRO-Ruby staining. Abundance of protein was calculated relative to FANCB and normalized by molecular weight. It is expressed as the mean +/– S.D. from two independent purifications including the preparation in Figure 1B.

SUPPLEMENTAL EXPERIMENTAL PROCEDURES

Purification of UBE2T

Chicken *UBE2T* was cloned from a chicken (DT40) cDNA library using adapted primers (5′-AAGTT CTGTT TCAGG GCCCG ATGCA GAGAG CATCG CGGC-3′ and 5′-ATGGT CTAGA AAGCT TTAGG GGTCC AGGCG AGATT TC-3′) into the pOPINJ vector using an InFusion cloning kit (Clontech) according to the manufacturer's protocol.

pOPINJ-*GgUBE2T* was transformed into BL21-DE3 pLacI cells and grown in LB supplemented with ampicillin and chloramphenicol. Expression was induced at OD_{600}=0.6 with 0.3 mM IPTG and grown at 22 °C overnight. Cells were harvested by centrifugation (4000 rpm, 4 °C, 25 min).

The cell pellet was resuspended in lysis buffer (50 mM Tris pH 8.0, 1 mM EDTA, 150 mM NaCl, 2 mM DTT) with 10 µg/ml DNase, 0.25 mg/ml lysozyme, 1 mM PMSF and Roche protease inhibitor cocktail. The suspension was rotated at 4 °C for 15 min and supplemented with $MgCl_2$ to 10 mM followed by rotation at 4 °C for 15 min. The lysate was sonicated on ice and supplemented with Igepal CA-630 to 0.1% followed by rotation at 4 °C for 15 min. The lysate was cleared by centrifugation (14 000 rpm, 4 °C, 30 min). The supernatant was incubated for 2 h at 4 °C with Glutathione Sepharose 4B beads (GE Healthcare Life Sciences) pre-equilibrated with lysis buffer. Beads were washed in a PD-10 column (GE Healthcare) with 30 bed volumes of lysis buffer with 0.1% Igepal CA-630, 60 bed volumes of lysis buffer with 0.5 M NaCl and 0.1% Igepal CA-630 and finally 60 bed volumes of cleavage buffer (50 mM Tris pH 7.4, 150 mM NaCl, 1 mM EDTA, 1 mM DTT, 0.1% Igepal CA630). Beads were transferred to a 2 ml collection tube and incubated with PreScission protease at 4 °C overnight with rotation to cleave the GST moiety, leaving a glycine-proline N-terminal extension. Eluted protein was collected by centrifugation through a Proteus mini clarification spin column (Generon). The flow-through, containing cleaved UBE2T, was applied to a S100 gel filtration column in buffer containing 50 mM HEPES pH 8.0, 150 mM NaCl, 10% glycerol and 5 mM β-mercaptoethanol and fractions containing pure *Gg*UBE2T were pooled and concentrated in an Amicon Ultracel-15 10 K MWCO centrifugal filter unit to 2 mg/ml (Figure S1A).

Purification of UBE2W

Chicken *UBE2W* was cloned from a commercially synthesised construct (IDT) using adapted primers (5′- AAGTT CTGTT TCAGG GCCCG ATGGC GTCCA TGCAG AAAAG-3′ and 5′- ATGGT CTAGA AAGCT TTATC AACAT GTGTC ATCAT GATAC C-3′) into the pOPINJ vector using an InFusion cloning kit (Clontech) according to the manufacturer's protocol.

pOPINJ-*GgUBE2W* was transformed into Rosetta2 pLysS cells and grown in 2XTY media supplemented with 1% glucose, 1 mM $MgSO_4$, ampicillin and chloramphenicol. Expression was induced at OD_{600}=2 with 1 mM IPTG at 30 °C for 2 h. Cells were harvested by centrifugation at (4000 rpm, 4 °C, 25 min).

The cell pellet was resuspended in lysis buffer (50 mM HEPES pH 8.0, 1 mM EDTA, 200 mM NaCl, 5 mM β-mercaptoethanol, 10% glycerol) supplemented with 0.25 mg/ml lysozyme, 1 mM PMSF, 2 mM benzamidine-HCl and Roche protease inhibitor cocktail. The lysate was sonicated on ice and supplemented with Igepal CA-630 to 0.1% followed by rotation at 4 °C for 15 min. The lysate was cleared by centrifugation (19 000 rpm, 4 °C, 25 min). The supernatant was incubated for 2 h at 4 °C with Glutathione Sepharose 4B beads pre-

equilibrated with lysis buffer. Beads were washed in a PD-10 column with 30 bed volumes of lysis buffer, 60 bed volumes of lysis buffer with 0.5 M NaCl and 60 bed volumes of cleavage buffer (50 mM HEPES pH 8.0, 150 mM NaCl, 1 mM EDTA, 10 mM β-mercaptoethanol). Beads were transferred to a 2 ml collection tube and incubated with PreScission protease at 4 °C overnight with rotation to cleave the GST moiety, leaving a glycine-proline N-terminal extension. Eluted protein was collected by centrifugation through Proteus mini clarification spin column (Generon). The flow-through, containing cleaved UBE2W was applied to a S75 gel filtration column in buffer containing 50 mM HEPES pH 8.0, 150 mM NaCl, 10% glycerol and 5 mM β-mercaptoethanol and fractions containing pure *Gg*UBE2W were pooled and concentrated in an Amicon Ultracel-15 10 KMWCO centrifugal filter unit to 2 mg/ml (Figure S1A).

Purification of FANCL

Sf9 cells were infected with a baculovirus encoding chicken GST-FANCL as previously described (Alpi et al., 2008). Cells were resuspended in lysis buffer (50 mM HEPES pH 8.0, 0.5 M NaCl, 10% glycerol, 1 mM EDTA, 0.1% Igepal CA-630, 1 mM PMSF, 5 mM β-mercaptoethanol and Roche protease inhibitor cocktail). The lysate was sonicated on ice and cleared by centrifugation (18 000 rpm, 4 °C, 45 min). The supernatant was incubated for 2 h at 4 °C with Glutathione Sepharose 4B beads pre-equilibrated with lysis buffer. Beads were washed in a PD-10 column with 30 bed volumes of lysis buffer, 60 bed volumes of lysis buffer with 0.5 M NaCl and 60 bed volumes of cleavage buffer (50 mM HEPES pH 8.0, 150 mM NaCl, 1 mM EDTA, 10 mM β-mercaptoethanol). Beads were transferred to a 2 ml collection tube and incubated with PreScission protease at 4 °C overnight with rotation to cleave the GST moiety. Eluted protein was collected and dialysed against 50 mM HEPES pH 8.0, 150 mM NaCl, 10% glycerol and 5 mM β-mercaptoethanol (Figure S1E).

Purification of FANCI and FANCD2

Constructs encoding chicken FANCI, FANCI^{K525R}, FANCD2 and FANCD2^{K563R} were a kind gift of H. Kurumizaka (Waseda University, Tokyo, Japan) and M. Takata (Kyoto University, Japan) and they were purified essentially as previously described (Sato et al., 2012). Briefly, proteins were expressed in Rosetta2-pLys cells and lysed in 50 mM HEPES pH 8.0, 0.5 M NaCl, 10% glycerol, 12 mM imidazole, 5 mM β-mercaptoethanol, 0.1% Igepal CA-630, 0.25 mg/ml lysozyme, 1 mM PMSF and protease inhibitor cocktail. Lysates were sonicated on ice, cleared by centrifugation (18 000 rpm, 4 °C, 45 min) and filtered through a 0.45 μM membrane. Proteins were purified using HisTrap, Heparin and Resource Q columns. Protein was loaded onto a Superdex S200 gel filtration column and separated by size exclusion in 20 mM HEPES pH 8.0, 200 mM NaCl, 10% glycerol and 5 mM β-mercaptoethanol. Fractions containing pure protein were pooled and concentrated in an Amicon Ultracel-15 100 KMWCO centrifugal filter unit to ~3 mg/ml (Figure S1C). His-tags were retained on proteins for immunodetection.

Purification of FANCB–FANCL–FAAP100 complex

cDNAs encoding full length *Gallus gallus* FANCB, FANCL and FAAP100 were synthesised (GeneArt). FANCB contained a C-terminal extension containing a PreScission protease site and StrepII tag. Both FANCL and FAAP100 were untagged. For protein expression, all genes

were cloned in to the MulitBac expression system and constructs generated as per manufacturers protocols (Bieniossek et al., 2012; 2008).

Briefly, *FANCB* and *FANCL* were subcloned via *Bam*HI and *Xba*I into the vector pIDC to generate pIDC-*FANCB*-SII and pIDC-*FANCL*. *FAAP100* was subcloned via *Bam*HI and *Xba*I into the vector pACEBac1 to generate pACEBac1-FAAP100. *FANCL* was subcloned from pIDC-*FANCL* as a *Bst*XI-PI-*Sce*I fragment into the PI-*Sce*I site in pIDC-*FANCB*-SII to generate pIDC-*FANCB*-SII**FANCL*. pIDC-*FANCB*-SII**FANCL* and pACEBac1-*FAAP100* were then fused using Cre recombinase (NEB) to generate pACEBac1-*FAAP100**pIDC-*FANCB*-SII**FANCL* (henceforth described as B–L–100). All constructs were confirmed by sequencing.

The B–L–100 construct was transformed under multiple antibiotic selection into DH10Bac *E. coli* competent cells containing the EmBacY expression bacmid. The purified bacmid was transfected into *Sf*9 cells in SF900-II medium (Invitrogen) using FuGene HD transfection reagent (Promega). Infection was monitored by YFP expression. A third generation amplification of the baculovirus was used to infect *Sf*9 cells for protein production.

At 72 hours post-infection, *Sf*9 cells were resuspended in lysis buffer (100 mM HEPES pH 7.0, 250 mM NaCl, 5% glycerol, 0.5 mM TCEP, 1mM benzamidine and Roche protease inhibitor cocktail). The lysate was sonicated on ice and cleared by centrifugation (19 000 rpm, 4 °C, 45 min). The supernatant was loaded onto a Streptactin affinity column equilibrated with wash buffer (lysis buffer without glycerol) and eluted with wash buffer supplemented with 6 mM desthiobiotin. Peak fractions were pooled and loaded on to a HiTrap HP SP column and eluted over a salt gradient at around 500 mM NaCl. Subsequently, peak fractions were loaded onto a Superose 6 XK16/70 gel filtration column and separated by size exclusion in 50 mM HEPES pH 8.0, 200 mM NaCl, 10% glycerol and 0.5 mM TCEP. Fractions containing pure protein were pooled and concentrated in an Amicon Ultracel-15 100 KMWCO centrifugal filter unit to ~6 mg/ml. The complex contained all three components in unit stoichiometry as assessed by SDS-PAGE followed by SYPRO-Ruby staining. Protein concentration was determined by Nanodrop ($A_{280\ nm}$ and calculated extinction coefficients) and subsequently checked by SDS-PAGE followed by Coomassie-staining to ensure FANCL in the B–L–100 complex was equimolar to isolated FANCL for activity assays.

DNA substrates

The DNA substrates were prepared by annealing HPLC-purified oligonucleotides resuspended in 20mM Tris pH 8.0, 50mM NaCl as previously described (Sato et al., 2012), for ssDNA (P1); dsDNA (P1, P7); splayed arm (P1, P2); 5' flapped DNA (P1, P2, P3); 3' flapped (P1, P2, P4); static fork (P1, P2, P3, P4); Holliday junction (P1, P2, P5, P6); and polyT (P8) substrates:

Primer	Sequence
P1	ATCGATGTCTCTAGACAGCTGCTCAGGATTGATCTGTAATGGCCTGGGA
P2	GTCCCAGGCCATTACAGATCAATCCTGAGCATGTTTACCAAGCGCATTG
P3	CAATGCGCTTGGTAAACA
P4	GCTGTCTAGAGACATCGAT
P5	TGATCACTTGCTAGCGTCGCAATCCTGAGCAGCTGTCTAGAGACATCGA
P6	CCAATGCGCTTGGTAAACATGCTCAGGATTGCGACGCTAGCAAGTGATC
P7	TCCCAGGCCATTACAGATCAATCCTGAGCAGCTGTCTAGAGACATCGAT
P8	TTTTTTTTTTTTTTTTTTTTTTTTTTTTTTTTTTTTTTTTTTTTTTTTTT

Cell Lines

DT40 cells were cultured in RPMI-1640 medium with L-glutamine (Gibco) supplemented with 7 % fetal bovine serum, 3 % chicken serum, 10 μM β-mercaptoethanol and penicillin-streptomycin mix (Gibco), at 37 °C in a humidified atmosphere with 5 % CO_2.

The Δ*FANCB* cell line was generated as follows: The targeting construct for the disruption of the last 3 exons of *Gallus gallus FANCB* (exons 7, 8 and 9; Ensembl gene ID ENSGALG00000016569, chromosome 1) was assembled by cloning the 5′ homology arm as a 3.1kb *Xho*I-*BamH*I fragment and the 3′ homology arm as a 5.4kb *BamH*I-*Spe*I fragment in pBluescript (Figure 1B). Homology arms were amplified from DT40 genomic DNA by PCR using *LA Taq* (TaKaRa, Japan), and the following oligonucleotides: 5′ arm forward 5′-CC*GTCGAC*GGGTTATCTTGGTAGCTTCAGTACCAGTCC (*Sal*I adapted); 5′ arm reverse 5′-CC*GGATCC*TCCAAGTTAATTCAGAGGCCGT GCAGTGTG (*BamH*I adapted); 3′arm forward 5′-CC*GGATCC*TATGTCACTT ATTTTATAGCTCAGATATT TGTAG (*BamH*I adapted); 3′arm reverse 5′-CC*ACTAGT*AAAACAAACAAAAAAACCCTAACATTCATT CCTC (*Spe*I adapted). Drug resistance marker cassettes for histidinol and blasticidin were cloned as *BamH*I inserts. The construct was linearized by *Not*I restriction digest prior to transfection. Drug resistant clones were screened by Southern blot after *BamH*I restriction digest of genomic DNA (Figures 1B and 1C). The probe fragment was amplified from DT40 genomic DNA by PCR using *LA Taq* (TaKaRa, Japan), and the following oligonucleotides: 5′-GCAGTGTTGCTTCCTTGCTGAGATCTCTGT; 5′-AAACAAAGCAAATTAAAGTGGA GGCTT; and then subcloned into pCR®2.1-TOPO® vector. *EcoR*I restriction digest of the vector releases a 1.3 kb fragment used for ^{32}P labelling and hybridisation. The probe used for Southern blot screening of drug-resistant clones hybridizes with either a 18 kb or 23 kb band in the wild type allele, due to a *BamH*I restriction fragment length polymorphism in the genomic locus of *FANCB*. The insertion of the resistance marker introduces a new *BamH*I site, which converts the wild type band into a 5.6 kb band in the targeted allele (Figures 1B and 1C).

The plasmid encoding chicken *FANCB* with a C-terminal GS tag was constructed as follows: The C-terminal GS tag from pGA1-C-GStag (synthesised by GeneArt) was sub-cloned as a *Xba*I-*Not*I fragment into *Nhe*I-*Not*I downstream of a linker sequence in pGA14-

linker (synthesised by GeneArt) to generate pGA1-link-C-GS comprising a linker peptide (GGGSGGGTGGGSGGG) upstream of a Streptavidin-Binding Peptide (SBP) tag, TEV cleavage site and two protein-G IgG-binding moieties. The linker-GS fragment was subcloned as an *Xba*I-*Not*I fragment at the 3′ end of *FANCB* cut with *Nhe*I and *Not*I in pCR2.1-FANCB cDNA. The complete *FANCB*-linker-GS fragment was subcloned as a *Spe*I fragment into the *Nhe*I site of pExpress-loxPuro and the orientation confirmed by sequencing.

The ΔB/B-GS cell line was generated by stably transfecting the GS-tagged *FANCB* cDNA into the *FANCB*-deficient (ΔB) DT40 line. DT40 cells were transfected using electroporation with a BioRad GenePulser II at 950 μF and 250 volts and selected in media supplemented with 0.5 μg/ml puromycin. Drug-resistant clones were screened by Western blot for expression of the GS-tagged FANCB protein. The ΔB/B-GS/ΔC cell line was generated by deleting *FANCC* as previously described (Niedzwiedz et al., 2004). Positive clones were confirmed by PCR amplification over the disrupted exon 2 and deleted exon 3 with oligonucleotides: 5′-GAGGA AATTA CTTAA GCTTT AACT-3′ and 5′-CTACA ACAAA GGGAT TCCAA CAAA-3′.

The targeting construct for the disruption of exon 4 of *GgUBE2W* (Ensembl gene ID ENSGALG00000019468, chromosome 2), containing the catalytic residue C91 in the UBC domain, was assembled by cloning the 5′ homology arm as a 4 kb *Not*I-*BamH*I fragment and the 3′ homology arm as a 4.4 kb *BamH*I-*Xho*I fragment into pBluescript. Drug resistance marker cassettes for puromycin, blasticidin and histidinol were cloned as *BamH*I inserts. The construct was linearized by *Not*I restriction digest prior to transfection. Three sequential rounds of targeting were performed, as chromosome 2 in DT40 is triploid. Drug-resistant clones were screened by Southern blotting after *BamH*I-*Kpn*I restriction digest of genomic DNA and PCR. The probe used for Southern blotting screening of drug-resistant clones hybridises with a 7.7 kb band in the wild type allele. The insertion of the resistance marker introduces a new *BamH*I site, which converts the wild type band into a 4 kb knock out band in the targeted allele. Homology arms were amplified from DT40 genomic DNA by PCR using *LA Taq* (TaKaRa, Japan), and the following oligonucleotides: 5′ arm forward 5′-AAAGACATTG AGCCGTTGGAGTGG; 5′ arm reverse 5′-GAAT ATTGTCACCAGTAAACATGA; 3′arm forward 5′-TTTCACCCATTCCAAGCTGGGATTTAT; 3′ arm reverse 5′-CCCTGGTACAATGTCCATTGC. The probe fragment was amplified from DT40 gDNA by PCR using *LA Taq* (TaKaRa, Japan), and the following oligonucleotides: forward 5'-CTCATC TGTCTCACTTGCAACTGATGT; reverse 5′-TTCCTGGAGCACCTTCCATGTCTACAA; and then subcloned in pCR®2.1-TOPO® vector. *EcoR*I restriction digest of the vector releases a 0.9 kb fragment used for ^{32}P labelling and hybridization. Targeting of the *GgUBE2W* locus was confirmed by PCR amplification of the deleted exon 4 with oligonucleotides: 5′-TCATATCT GTTTGTCCATTCT and 5′-TTCTTTGCAGCTGGAAAGCAT.

The constructs for stable expression of GFP-USP1(WT) and GFP-USP1(C92S) have been previously described(Oestergaard et al., 2007).

Δ*FANCC* (Niedzwiedz et al., 2004), Δ*FANCG* (Niedzwiedz et al., 2004), Δ*FANCL* (Alpi et al., 2007), Δ*FAAP100* (Ling et al., 2007), Δ*FANCM* (Mosedale et al., 2005), Δ*USP1* (Oestergaard et al., 2007) and Δ*UBE2T* (Alpi et al., 2007) cell lines are previously published. For the Δ*FANCA* cell line, a targeting construct for the disruption of exons 7, 8 and 9 of *GgFANCA* (Ensembl gene ID ENSGALG00000000516, chromosome 11) was assembled by cloning the 5′ homology arm as a 2 kb *Spe*I-*Bgl*II fragment and the 3′ homology arm as a 2 kb *Bgl*II-*Not*I fragment in pBluescript. Drug resistance marker cassettes for blasticidin and

puromycin were cloned as *BamH*I inserts in the *Bgl*II site. The Δ*FANCF* cell line was generated as follows: The targeting construct for the disruption of the single exon of chicken *FANCF* (Ensembl gene ID ENSGALG00000003660, chromosome 5) was assembled by cloning the 5′ homology arm as a 2 kb *Sal*I-*BamH*I fragment and the 3′ homology arm as a 2 kb *BamH*I-*Not*I fragment in pBluescript. Drug resistance marker cassettes for blasticidin and puromycin were cloned as *BamH*I inserts. The construct was linearized by *Not*I restriction digest prior to transfection. Drug resistant clones were screened by Southern blot after *Nhe*I and *Spe*I restriction digest of genomic DNA. The probe used for Southern blot screening of drug-resistant clones is a 1 kb fragment that hybridises with a 5.3 kb band in the wild type allele. The insertion of the resistance marker removes a genomic *Nhe*I site, which converts the wild type band into a 7.5 kb (blasticidin) or 4.5 kb (puromycin) knock out band in the targeted allele, following the insertion of a new *Nhe*I site.

Human, mouse and hamster cells were maintained in DMEM medium (Gibco) with 10% fetal bovine serum and penicillin-streptomycin mix.

Fanconi Anaemia Core Complex Purification

For purification, ΔB/B-GS or ΔB/B-GS/ΔC DT40 cells were grown to confluency in 2 L roller bottles and harvested by centrifugation (2 000 rpm, 4 °C, 20 min). The cell pellet was washed twice in ice-cold PBS supplemented with a protease inhibitor cocktail. The tandem affinity purification scheme is modified from the protocol described by Bürckstümmer et al. (2006). The cell pellet (~3 g (wet) / 2-4 L culture) was resuspended in ice-cold GS buffer (50 mM HEPES pH 8.0, 150 mM NaCl, 5 % glycerol, 0.1 % Igepal CA-630, 1.5 mM $MgCl_2$, 25 mM NaF, 2 mM Na_3VO_4, 40 mM β-glycerophosphate, 1 mM PMSF, 10 mM β-mercapto-ethanol, PhosSTOP inhibitor cocktail (Roche) and protease inhibitor cocktail). The cell suspension was passed through 19 G and 25 G needles multiple times and incubated on ice for 20 min. The lysate was clarified by high-speed centrifugation (13 200 rpm, 4 °C, 40 min). All subsequent steps were performed at 4 °C unless otherwise stated.

For the first affinity step (via the protein-G IgG binding moieties in the GS tag), the lysate was incubated with IgG-agarose beads (Sigma) at 4 °C for 2 h with gentle rotation in a sealed PD-10 column. The beads were allowed to settle and the flow-through was collected by gravity. The beads were washed extensively with 30 bed volumes of wash buffer (50 mM HEPES pH 8.0, 150 mM NaCl, 10 % glycerol, and phosphatase/protease inhibitors) and 30 bed volumes of wash buffer supplemented with 5 mM β-mercaptoethanol. A tobacco etch virus (TEV) protease site upstream of the protein-G tag was cleaved to elute the protein complex from the beads: The beads were transferred to a LoBind 1.5 ml tube (Eppendorf) and incubated with 100 µg TEV protease at 16 °C for 2 h with gentle agitation in a thermomixer. The cleavage reaction was transferred to a Proteus clarification mini spin column (Generon) and the supernatant collected by centrifugation (10 000 rpm, 4 °C, 5 min).

For the second affinity step, (via the SBP tag), the supernatant was incubated with Ultralink Streptavidin Plus Resin (Pierce) in a 1.5 ml tube with gentle rotation for 1 h. The binding reaction was transferred to a Poly-Prep chromatography column (BioRad) and washed with 100 bed volumes of wash buffer supplemented with 5 mM β-mercaptoethanol.

To mildly elute the SBP tag from streptavidin, the resin was transferred to a 1.5 ml tube and incubated with one bed volume of wash buffer supplemented with 3 mM β-mercapto-ethanol and 6 mM D-biotin (Sigma) on ice for 20 min with gentle agitation. The elution reaction was transferred to a Proteus mini clarification spin column and the purified FA core

complex was collected by centrifugation (10 000 rpm, 4 °C, 5 min). The complex was resolved by SDS-PAGE and analysed by Coomassie, silver (Sigma) or SYPRO-Ruby (Lonza) staining. Gels were imaged on a BioRad ChemiDoc XRS+ imager and analyses performed using ImageLab 3.0 software (BioRad laboratories). Bands were excised, digested and identified by MS/MS (data not shown) using multiple independent purifications. The major species for each band is labelled on Figure 2B. The band containing tubulin also contains small amounts RuvBL1/2. Purified complex was used fresh, immediately after purification, for downstream biochemical analyses. A titration against known concentrations of recombinant FANCL was used to approximate the concentration of prepared FA core complex on SDS-PAGE. We estimate that from an ~3 g DT40 cell pellet (2-4 L cell culture), we purify ~5 μg FA core complex.

Whole cell extract preparation

Cells were washed in PBS, harvested by trypsinization and collected by centrifugation. Whole cell extracts (WCEs) were prepared by lysis in ice-cold NET-N buffer (50 mM HEPES pH 8.0, 150 mM NaCl, 1 mM EDTA, 0.5 % Igepal CA-630) or ice-cold RIPA buffer (50 mM Tris-HCl pH 7.4, 150 mM NaCl, 0.5 % sodium deoxycholate, 0.1 % SDS and 1 % Igepal CA-630 (Sigma)). Buffers were freshly supplemented with 1 mM DTT, 1 mM PMSF, 2 mM Na_3VO_4, 25 mM NaF, 40 mM β-glycerophosphate, 1 X Halt Phosphatase Inhibitor Cocktail (Pierce) and a Protease Inhibitor Cocktail (Roche)). Lysates were cleared by centrifugation (13 200 rpm, 4 °C, 25 min). Protein concentration was quantified using the Bicinchoninic Assay (BCA) (Thermo Scientific). WCEs (30 μg per sample) were analyzed by Western blotting.

Cellular fractionation

DT40 cells were fractionated into a cytosol/nucleosoluble fraction (S) and a chromatin/nuclear matrix fraction (P) essentially as previously described (Kim et al., 2008). Briefly, cells were lysed on ice in CSK buffer (20 mM PIPES pH 7.0, 100 mM NaCl, 340 mM sucrose, 1 mM EDTA, 0.3 % Triton X-100, 25 mM NaF, 1 mM Na_3VO_4, 1 mM PMSF, 1 mM DTT, protease inhibitor cocktail (Roche) and PhosSTOP (Roche)) and resuspended by gently pipetting. The supernatant (S) was collected by low speed centrifugation (1300 x g, 5 min, 4 °C). The pellet was washed again in CSK buffer and resuspended on ice in CSK buffer supplemented with 1 % Triton X-100, 0.5 % sodium deoxycholate, 0.1 % SDS and 0.5 M NaCl. Samples were vigorously vortexed, sonicated and the supernatant (P) collected by high-speed centrifugation (15 000 rpm, 1 h, 4 °C). Protein was quantitated, resolved by SDS-PAGE and analyzed by Western blotting.

Western blotting

Samples were resolved by SDS-PAGE on 4–12 % Bis-Tris gels or 3–8 % Tris-Acetate gels (Life Technologies) and transferred to PVDF membranes (Millipore) in 1 X transfer buffer (Life Technologies) supplemented with 20 % methanol before blotting with the appropriate antibodies: HRP-conjugated anti-HA (F-7, Santa Cruz Biotechnology), HRP-conjugated anti-HIS (H-3, Santa Cruz Biotechnology), anti-β-actin (AC-15, Sigma), anti-PCNA (PC10, Santa Cruz Biotechnology), anti-GFP (FL, Santa Cruz Biotechnology), anti-chicken FANCG (Alpi et al., 2007), anti-chicken FANCM (Mosedale et al., 2005) or anti-chicken FANCD2 (gift of Michael Hodskinson, MRC LMB). The protein G moiety in the GS-tag is detected with the

antibody against FANCD2 in Figures 1D and 5A. Quantification of FANCD2 ubiquitination was performed using ImageJ software.

Ubiquitination assay

Reactions in 10–20 μl volumes were performed in a buffer comprising 50 mM HEPES pH 7.5, 64 mM NaCl, 4 % glycerol, 5 mM $MgCl_2$, 2 mM ATP and 0.5 mM DTT. In all reactions, 75 nM E1 (Boston Biochem) and 1 μM E2 (UBE2T or UBE2W) enzymes, 1 μM substrate (FANCI, $FANCI^{K525R}$, FANCD2, $FANCD2^{K563R}$), 50 μM 5′-flapped DNA (nucleotide concentration) and 20 μM HA–ubiquitin (Boston Biochem) were used unless otherwise stated. E3 (FANCL or FA core complex) concentrations are indicated in figures. Optimal E2 and FANCL concentrations were determined by titrations.

All reactions were incubated at 30 °C for 90 min (or for indicated times as part of a time course) and quenched by the addition of LDS sample buffer (Life Technologies). Samples were analysed by Western blotting.

MMC treatment

Cellular sensitivity of DT40 cells was assayed by a colony formation assay as previously described (Niedzwiedz et al., 2004). For induction of FANCD2 monoubiquitination, DT40 and human cells were treated for 18 h with 50 ng/ml MMC. Rodent cells were treated for 18 h with 333 ng/ml MMC.

Supplemental References

Bieniossek, C., Imasaki, T., Takagi, Y., and Berger, I. (2012). MultiBac: expanding the research toolbox for multiprotein complexes. Trends Biochem. Sci. *37*, 49–57.

Bieniossek, C., Richmond, T.J., and Berger, I. (2008). MultiBac: multigene baculovirus-based eukaryotic protein complex production. Curr Protoc Protein Sci *Chapter 5*, Unit5.20.

Gallmeier, E., Calhoun, E.S., Rago, C., Brody, J.R., Cunningham, S.C., Hucl, T., Gorospe, M., Kohli, M., Lengauer, C., and Kern, S.E. (2006). Targeted Disruption of FANCC and FANCG in Human Cancer Provides a Preclinical Model for Specific Therapeutic Options. Gastroenterology *130*, 2145–2154.

Koomen, M. (2002). Reduced fertility and hypersensitivity to mitomycin C characterize Fancg/Xrcc9 null mice. Hum. Mol. Genet. *11*, 273–281.

Nomura, Y., Adachi, N., and Koyama, H. (2007). Human Mus81 and FANCB independently contribute to repair of DNA damage during replication. Genes Cells *12*, 1111–1122.

Scaglione, K.M., Basrur, V., Ashraf, N.S., Konen, J.R., Elenitoba-Johnson, K.S.J., Todi, S.V., and Paulson, H.L. (2013). The Ubiquitin-conjugating Enzyme (E2) Ube2w Ubiquitinates the N Terminus of Substrates. J. Biol. Chem. *288*, 18784–18788.

Tatham, M.H., Plechanovová, A., Jaffray, E.G., Salmen, H., and Hay, R.T. (2013). Ube2W conjugates ubiquitin to α-amino groups of protein N-termini. Biochem. J. *453*, 137–145.

Wilson, J.B., Johnson, M.A., Stuckert, A.P., Trueman, K.L., May, S., Bryant, P.E., Meyn, R.E., D'Andrea, A.D., and Jones, N.J. (2001). The Chinese hamster FANCG/XRCC9 mutant NM3 fails to express the monoubiquitinated form of the FANCD2 protein, is hypersensitive to a range of DNA damaging agents and exhibits a normal level of spontaneous sister chromatid exchange. Carcinogenesis *22*, 1939–1946.
